# Advances in AAV technology for delivering genetically encoded cargo to the nonhuman primate nervous system

**DOI:** 10.1016/j.crneur.2023.100086

**Published:** 2023-04-07

**Authors:** Lillian J. Campos, Cynthia M. Arokiaraj, Miguel R. Chuapoco, Xinhong Chen, Nick Goeden, Viviana Gradinaru, Andrew S. Fox

**Affiliations:** a Department of Psychology and the California National Primate Research Center, University of California, Davis, CA, 05616, USA; b Division of Biology and Biological Engineering, California Institute of Technology, Pasadena, CA, 91125, USA; c Aligning Science Across Parkinson's (ASAP) Collaborative Research Network, Chevy Chase, MD, 20815, USA; d Capsida Biotherapeutics, Thousand Oaks, CA, 91320, USA

**Keywords:** Nonhuman primate, Adeno-associated viral vectors, Capsid variants, Enhancers, Brain, Gene delivery

## Abstract

Modern neuroscience approaches including optogenetics, calcium imaging, and other genetic manipulations have facilitated our ability to dissect specific circuits in rodent models to study their role in neurological disease. These approaches regularly use viral vectors to deliver genetic cargo (e.g., opsins) to specific tissues and genetically-engineered rodents to achieve cell-type specificity. However, the translatability of these rodent models, cross-species validation of identified targets, and translational efficacy of potential therapeutics in larger animal models like nonhuman primates remains difficult due to the lack of efficient primate viral vectors. A refined understanding of the nonhuman primate nervous system promises to deliver insights that can guide the development of treatments for neurological and neurodegenerative diseases. Here, we outline recent advances in the development of adeno-associated viral vectors for optimized use in nonhuman primates. These tools promise to help open new avenues for study in translational neuroscience and further our understanding of the primate brain.

## Highlights

• Nonhuman primates are a valuable model for study of human disease.
• Technical hurdles have impeded translation of gene delivery from mice to primates.
• Capsid engineering allows for systemic delivery to primate nervous system.
• Identification of gene regulatory elements may further enable cell-type specificity.

## Graphical abstract

**Figure:**
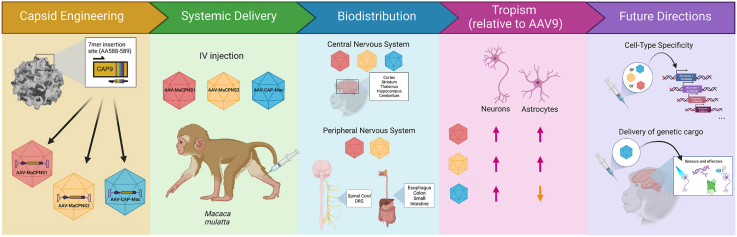

## 1 Nonhuman primate as a valuable model for study of human diseases

A major goal of modern neuroscience is to inform our understanding of the human condition and brain-based disorders. However, this requires better comprehension of the distributed anatomical and molecularly diverse pathways, and functional circuits underlying disease. Recent advances in genetic technologies have made it possible to control and image neuronal circuits in living animals, through the delivery of various effectors, sensors, and reporters to the brain (Boyden et al., 2005; Fenno et al., 2011; Wang et al., 2019; Yang and Yuste, 2017). This breakthrough in technology has advanced our understanding of neural circuits, cell-types, molecules, neurotransmitters, and gene regulatory elements that work together to contribute to the progression of disease (e.g.(Coley et al., 2021; Cummings and Clem, 2020; Fadok et al., 2018; Pignatelli and Beyeler, 2019; Xu et al., 2019)). For example, research on anxiety-relevant circuits has leveraged optical control of specific cell-types (e.g. somatostatin and corticotrophin-releasing hormone expressing cells) and their projections to threat-relevant regions (e.g. central amygdala to periaqueductal gray interneurons) in order to elucidate multiple distinct mechanisms that underlie specific aspects of threat responding behavior (Ciocchi et al., 2010; Fadok et al., 2017; Holley and Fox, 2022; Tovote et al., 2016). This work has far-reaching implications for our understanding of anxiety disorders, by identifying multiple distinct mechanisms that likely contribute to differences in symptomatology. Similarly, in basic research studies of the mechanisms relevant to neurodegenerative diseases like Parkinson's, optical inhibition of cells in the subthalamic nucleus of parkinsonian rodents was sufficient to improve 6-hydroxydopamine-induced forelimb akinesia, opening the door to potential treatment avenues for patients with PD (Yoon et al., 2014). Unfortunately, these advances have not led to a large number of new and improved treatments for neurological diseases. This gap between rodent models of disease and translational outcomes is due, in part, to difficulty in validating the relevancy of these potential targets in human disease, as well as in understanding how these potential therapeutic compounds interact with their molecular targets in primates. Differences in evolutionary pressures have contributed to differences in brain structure and function across species, including the expansion of cortical regions during human evolution (Ahmed, 2018; Baker et al., 2020; Ménard et al., 2016; Pine et al., 2021; Preuss and Wise, 2022), potentially contributing to poor predictability of rodent disease models to the human condition. Nonhuman primates (NHPs) are well-suited to bridge this gap as their recent evolutionary divergence from a common ancestor have endowed them with many anatomical, physiological, and ethological similarities to humans (Kalin and Shelton, 2003; Öngür and Price, 2000; Petrides and Pandya, 1999, 2002; Phillips et al., 2014). This makes them well suited for anatomical and functional dissection in both the central nervous system (CNS) and peripheral nervous system (PNS), and as models for interrogation of potential therapeutic targets.

**Fig. 1:**
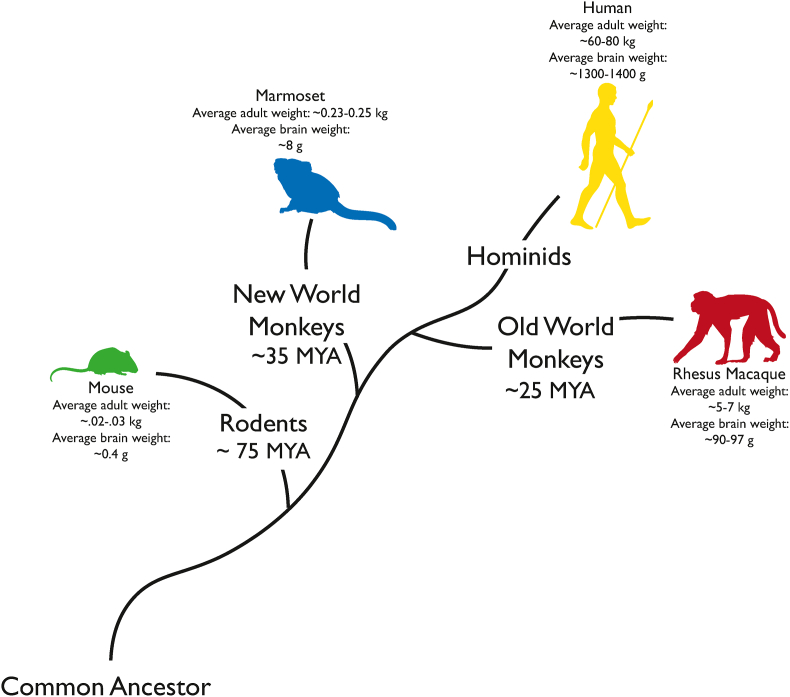
Evolutionary tree depicting the phylogentic relationship of common research species. Among these species, Old World Monkeys, like the rhesus macaque, are approximately more similar to humans than New World Monkeys and rodents.

Perhaps the most notable distinction between human and rodent brains is the expansion of neocortex during human evolution (Kaas, 2012; Öngür and Price, 2000; Petrides and Pandya, 1999, 2002; Pine et al., 2021). This expansion is often thought to have contributed to the many high order abilities and social complexities related to human uniqueness (Kaas, 2012; Smaers et al., 2011). Many studies in humans have shed light on the neuronal circuits associated with these abilities, however, due to the limitations of the available tools for the study of the human brain, a more comprehensive understanding of the underlying biology is still needed (Craig, 2009; Cristofori et al., 2019; Gläscher et al., 2012; Horga et al., 2014). To this end, NHPs are of particular importance. To briefly review, NHPs can be roughly broken down into various simian species, which include monkeys and apes, and prosimians, such as lemurs. Monkeys can be further divided into Old World (Catarrhini) and New World (Platyrrhini) monkeys (Welker, 2017). Marmosets (*Callithrix jacchus*), which diverged from the human lineage approximately 35 million years ago (MYA), and rhesus macaques (*Macaca mulatta),* which diverged from humans even more recently, approximately ∼25 MYA (Fig. 1), are the two most common NHPs used in research. It is this recent evolutionary divergence from a common ancestor that has made NHPs a valuable model in neuroscience, as they possess a highly elaborated prefrontal cortex, including a well-developed internal granular layer (Bernardi and Salzman, 2019; Öngür and Price, 2000; Petrides and Pandya, 1999, 2002). Because they are our phylogenetic neighbors, NHPs share many behavioral and anatomical features with humans (Kalin and Shelton, 2003; Öngür and Price, 2000; Petrides and Pandya, 1999, 2002; Phillips et al., 2014). For example, the ability to navigate social complexities has been hypothesized to be enabled by the evolutionary expansion of the primate prefrontal cortex (Dunbar and Shultz, 2007; Pine et al., 2021). Indeed, unlike many animals, both NHPs and humans have developed complex social behaviors that have helped them navigate the complexities of living in large social groups (Chang and Platt, 2014). These include prosocial behaviors (Miller et al., 2016), social imitation (Subiaul et al., 2004), and in New World Monkeys like marmosets, monogamy and infant rearing (Miller et al., 2016; Saito, 2015). The shared social repertoires between monkeys and humans have been helpful in studying the underlying biology of social behaviors (Chang and Platt, 2014; Ziegler, 2018). In addition to social behaviors, the phylogenetic proximity of Old World monkeys, like rhesus macaques, to humans provides an avenue to study the primate brain which has a similar structure and cytoarchitecture to the human brain (Öngür and Price, 2000; Petrides and Pandya, 1999, 2002). For this reason, primates can contribute to understanding human-specific cognitive functions like higher-order cognition, attention, and working memory (Brady and Hampton, 2018; Deaner and Platt, 2003; Dezfouli et al., 2021; Rich and Wallis, 2016; Snyder et al., 2021; Xie et al., 2022).

While the phylogenetic proximity of NHPs to humans has made them anatomically and behaviorally similar, it is also likely that throughout evolution, the composition and function of neuronal circuits have adapted based on the evolutionary pressures placed on the species (Katz and Harris-Warrick, 1999). That is to say, while rodents and humans may share basic organization of circuits, changes within these circuits can cause large and important changes in behaviors (Katz and Harris-Warrick, 1999). Indeed, recent studies have found that while there seems to be a strong evolutionary conservation of cell-types between rodents and primates, there are also a number of primate-specific cell-types distributed throughout the brain-in cortical and subcortical structures and in midbrain (Hodge et al., 2019; Kamath et al., 2022; Krienen et al., 2020; Schmitz et al., 2022). For example, von Economo neurons and fork neurons which were originally assumed to exist only in humans and great apes, have been identified in Old World Monkeys (Evrard, 2019). This discovery provides the opportunity to elucidate the function of these neurons, as they have been shown to have projections to regions often implicated in studies of psychological dysfunction in both humans and NHPs (Craig, 2009; Evrard, 2019; Fox et al., 2015). Similarly, primates have a larger diversity of interneurons (Fishell and Kepecs, 2020; Franjic et al., 2022; Krienen et al., 2020; Schmitz et al., 2022), which may impact circuit dynamics in ways that cannot be studied in rodent models. For example, researchers have identified a primate-specific striatal interneuron (i.e. GABAergic neurons that express *TAC3*), which is thought to have emerged through a developmental repurposing of dopaminergic periglomerular cells of the olfactory bulb (Krienen et al., 2020; Schmitz et al., 2022). Additionally, a novel excitatory cell type expressing neuropeptide Y (*NPY*) was recently discovered in the primate visual cortex (Wei et al., 2022). As we aim to further dissect and elucidate the distributed brain-body circuits related to human pathology, the ability to target and manipulate specific cells will become increasingly important as they may be key determinants in the emergence of brain based disorders in humans.

In brief, rodent models have been useful in understanding the molecular and cellular basis of diseases, due in large part to advances in molecular genetic technologies (Boyden et al., 2005; Fenno et al., 2011; Yang and Yuste, 2017). However, rodents and humans differ in physiology, anatomy, and social complexity. To this end, NHPs are of particular importance because of the recent evolutionary divergence between humans and primates that have made them similar to humans both biologically and socially. However, a critical component of translation includes the translation of the tools that have been commonplace in rodent neuroscience. In this review, we highlight the need for more efficient neurotropic AAVs that can be delivered systemically in NHPs, recently engineered capsid variants that can cross the blood brain barrier in NHPs, and advances made to target specific cell-types.

## 2 AAVs enable gene delivery and circuit interrogation

The ability to define, monitor, and manipulate a neural circuit requires precise delivery of reporters, sensors, and effectors to the individual circuit components (e.g. cell-types). Viral vectors such as herpes simplex virus (HSV), rabies, adenovirus, lentivirus (LV), and adeno-associated viruses (AAVs) have emerged as an effective tool for neuroscience in that they enable neuronal tracing and functional interrogation through the delivery of various transgenes (Davidson and Breakefield, 2003; Ghosh et al., 2020; Hui et al., 2022; Kristensson et al., 1982; Liu et al., 2022). Viral vectors are composed of: i) a capsid, an outer protein shell enclosing the genetic material and which determines the vector's tropism, or ability to infect different cell-types; ii) regulatory elements such as enhancers or promoters which restrict expression to specific cell or tissue types; and iii) a transgene (Bulcha et al., 2021). Transgenes include fluorescent proteins as genetic reporters for visualization, sensors for measuring neurotransmitter release (e.g., GCaMP, DLight, etc.), opsins and synthetic receptors for cellular manipulation (e.g., ChR2, DREADDs), and repair templates for CRISPR-Cas9 based gene editing and expression manipulation (e.g., using CRISPRa/i) (Boyden et al., 2005; Klapoetke et al., 2014; X. Li et al., 2005; Magnus et al., 2011; Patriarchi et al., 2018; Roth, 2016; Yim et al., 2020; F. Zhang et al., 2007). Such genetic tools have advanced our understanding of how various cell-types and specific circuits contribute to adaptive behaviors and emergent properties of the brain. Among the viral vectors, AAVs are considered to be the safest since they are non-pathogenic, and are naturally replication deficient i.e. they lack the genes necessary for replication and replicate only when co-infected with a helper virus (Buller et al., 1981; Rose and Koczot, 1972). In contrast, lentiviruses transduce cells with higher efficiency than AAVs but there is uncertainty surrounding their safety due to the possibility of random insertional mutagenesis (Zheng et al., 2018). This can affect the genetic code at the DNA insertion site, leading to adverse outcomes, including cancer (Zheng et al., 2018). Similarly, herpes viral vectors can cause strong inflammatory responses (Ghosh et al., 2020). These non-specific and adverse effects have precluded them from widespread use in NHPs (Tremblay et al., 2020). Additionally, AAVs are capable of transducing both dividing and post-mitotic cells such as neurons, and so are ideally suited for gene manipulation studies that require stable, long term transgene expression in cells that have already matured (Bartlett et al., 1998). For these reasons, recombinant AAVs (rAAVs) have become the viral vector of choice for *in vivo* gene therapy applications, with more than 285 registered clinical trials to date (Kuzmin et al., 2021; U.S. National Library of Medicine (www.clinicaltrials.gov)).

For the study and treatment of neurological and neurodegenerative diseases, widespread distribution of transgene expression could be transformative (Hadaczek et al., 2016; Muramatsu, 2010; Sun and Roy, 2021). For example, idiopathic Parkinson's disease is hypothesized to result from the aggregation of a protein called alpha-synuclein (α-Syn) first in the enteric nervous system, before it propagates up the vagus nerve to the basal forebrain, midbrain and ultimately the cerebral cortex (Braak et al., 2003). rAAVs can be used to deliver a pathogenic protein such as α-Syn to model PD in animals, and help tease apart the cell-types in the ENS and CNS that are susceptible to α-Syn pathology (Alam et al., 2022; Challis et al., 2020; Huntington and Srinivasan, 2021; Kirik and Björklund, 2003; Ulusoy et al., 2010). In such cases, widespread transgene expression is required. Conversely, the pathogenic protein can be silenced, or the disease phenotype may be reversed by delivering a therapeutic gene such as *GBA1*, which encodes the lysosomal enzyme Glucerebrosidase, and has been shown to reduce inflammation and aggregation of α-Syn in models of PD as well as Gaucher's disease-a lysosomal neurodegenerative disorder (Abeliovich et al., 2021; Sardi et al., 2011, 2013). The route of AAV administration, dose, age at the time of injection, and preexisting neutralizing antibodies against it in the host, are all key determinants of an AAV's safety, efficacy and tropism (Chan and Deverman, 2022). There are many advantages to direct in-brain injection. In fact, most studies to date have directly injected viruses into the brain to deliver genetic cargo to specific regions. This has been performed in animal models of PD to target the putamen or substantia nigra (Bartus et al., 2013; Christine et al., 2009; Kaplitt et al., 2007; Kells et al., 2012; Muramatsu, 2010). Despite its many advantages, which include relatively dense and robust expression surrounding the infusion site, targeting large, diffuse, or spatially distributed regions can require multiple injections. Thus, this route of administration is most suitable for localized targets (Wang, 2021). Covering entire brain regions remains a challenge due to the size of the primate brain and often requires multiple craniotomies. Systemic delivery of AAVs obviates the need for multiple direct injections and importantly reduces the health risks associated with extremely long and highly invasive surgeries. As a therapeutic approach, systemic administration via a single injection might be a safer alternative toward achieving brain wide gene transduction (Bourdenx et al., 2014; Kimura and Harashima, 2022). Ultimately, increased efficacy of BBB-crossing AAVs may be combined with other technologies to achieve localized expression. However, crossing the BBB, which acts as a gatekeeper by preventing toxins and pathogens in the systemic circulation from entering the CNS, is a major hurdle for efficient gene delivery.

To date, 13 distinct naturally-occurring or *wildtype* serotypes of AAVs, (AAV1-13) have been identified in humans and NHPs (Srivastava, 2016). Each of these serotypes differs in capsid structure and, therefore, tropism (Agbandje-McKenna and Kleinschmidt, 2012). The most commonly used serotypes in rodent research are AAV2, AAV5, AAV8, and AAV9, which transduce the CNS, although some transduce other organs as well (Aschauer et al., 2013). In NHPs, the most commonly used serotypes are AAV5 and AAV9 (Tremblay et al., 2020). AAV9 has been particularly widely studied because of its ability to cross the BBB and has been employed in several CNS-targeted gene therapies (Chan and Deverman, 2022; Chen et al., 2021; Foust et al., 2009; Song et al., 2022; Yang et al., 2014; Zhang et al., 2011). Efforts have also been made to characterize other serotypes that also have the capability to cross the BBB. For example, Gao et al. cloned and identified more than 100 novel rAAVs from human and NHP tissues (Gao et al., 2005; Gao et al., 2002). Among these, AAVrh8, AAVrh10 and AAVhu32 were found to cross the BBB with high efficiencies, similar to AAV9.

However, these AAVs, including AAV9, have limitations that have prevented their wider use. For instance, their cell-type tropism can vary across species. In neonatal mice and macaques, intravenously administered AAV9 transduces neurons preferentially, whereas in juvenile and adult mice and macaques, the tropism shifts toward astrocytes (Bevan et al., 2011; Dehay et al., 2012; Foust et al., 2009; Gray et al., 2011; Mattar et al., 2013; Samaranch et al., 2012). Moreover, AAV9 and the other BBB crossing serotypes mentioned above have a higher tropism for peripheral organs such as the liver than the brain (Gray et al., 2011; Zincarelli et al., 2008). This is especially concerning in large animals such as NHPs as they require large volumes of virus for systemic delivery and the high doses of AAV needed to achieve clinical relevance can lead to hepatotoxicity or sensory neuron toxicity (Hinderer et al., 2018). Additionally, humans as well as NHPs harbor neutralizing anti-AAV antibodies to certain wildtype AAV serotypes from pre-existing exposure or develop anti-AAV antibodies after therapeutic rAAV administration (Louis Jeune et al., 2013). This is a limiting factor for gene therapy applications where subsequent viral administration may be needed if the transgene expression wanes over time. Another important consideration is the ∼4.7 kb size limitation of the AAV vector genome, which is comparatively smaller than that of lentiviruses (∼9.7 kb) or herpes simplex virus (∼150 kb) (Kumar et al., 2001; Latchman, 2001; Sena-Esteves et al., 2000).

When designing a study, it is important to take these considerations into account. In studies of the primate brain, it is important to ensure that the target gene sequence is reliably expressed, to minimize off-target effects, and to ensure animal safety. This has led most studies to prefer AAVs. However, if the genetic cargo is larger than optimal for an AAV genome, researchers run the risk of lower transduction efficiency, affecting their ability to perform the desired manipulation (Wu et al., 2010). These cost-benefit calculations are study-specific and constantly changing. Development of cell-type specificity, as a function of the viral capsid or shortened enhancer and promoter cargo, as described below could mitigate off-target effect. In the following sections, we will discuss current efforts to develop novel systemic rAAV vectors with high transduction efficiency and optimized biodistribution, with minimal off-target delivery, and low immunogenicity for gene delivery to the NHP CNS (Challis et al., 2022; Chan and Deverman, 2022; Davidson et al., 2022).

## 3 Capsid engineering for systemic delivery to achieve tissue specific biodistribution in NHPs

The capsid of an AAV is its primary point of interaction with receptors on the host cell surface which enable the virus to be internalized, and ultimately deliver their genetic cargo to the cell nucleus (Challis et al., 2022; Li and Samulski, 2020). Because of this, the capsid structure of AAVs have been widely researched in order to determine the protein domains responsible for cellular receptor binding, and consequently the virus' tropism and efficacy (Challis et al., 2022; Lee et al., 2018; Li and Samulski, 2020). Capsid modification or engineering is one route toward altering an AAV's tropism and efficacy as several permissive sites for rational and random amino acid substitutions and insertion have been identified (Challis et al., 2022). Through capsid engineering, we can enhance and refine AAV tropisms, as well as identify novel AAV serotypes with improved BBB crossing properties.

Capsid engineering can be carried out either through rational design or directed evolution. Rational design capitalizes on the knowledge of existing AAV serotypes to systematically predict and refine virus function (Lee et al., 2018). On the other hand, directed evolution is a high-throughput approach that involves using a selection process to generate variants with the desired properties such as antibody neutralization and/or tissue and cell-type tropism. By iteratively creating many non-naturally occurring viral serotypes and selecting those with the desired tropism for the next round of evaluation, researchers have been able to discover novel capsid variants that have a higher transduction efficiency at lower titers or concentration (Chan et al., 2017; Deverman et al., 2016; Körbelin et al., 2016; Kumar et al., 2020; Nonnenmacher et al., 2021). For example, our group has developed the Cre recombination-based AAV targeted evolution (CREATE) selection method (Deverman et al., 2016). In brief, the CREATE method enables the recovery of capsid sequences that transduce Cre-expressing cell populations in transgenic mice. Wherever Cre is present, a library fragment adjacent to the cap gene is inverted. PCR-based amplification can then detect the sequences that have successfully transduced the target population. After multiple rounds of evolution, top performing capsids can then be selected and characterized. This method led to the identification of AAV-PHP.B and AAV-PHP.eB, which target the mouse CNS (Chan et al., 2017; Deverman et al., 2016). Both AAV-PHP.B and AAV-PHP.eB are derived from AAV9, with AAV-PHP.eB showing >50% transduction of cells across brain regions of C57BL/6J mice (Chan et al., 2017). However, the ability of these engineered variants to transduce cells in the CNS of other mouse strains or NHPs is dependent on the administration route and whether it can cross the BBB (Mathiesen et al., 2020). Indeed, IV administration of AAV-PHP.B in marmosets showed poor transduction of neurons and astrocytes similar to AAV9 (Matsuzaki et al., 2018). Furthermore, Hordeaux et al. reported that a higher dose (7.9E13 gc/kg) of the AAV-PHP.B vector resulted in acute toxicity in an IV-injected macaque (Hordeaux et al., 2018). Subsequent studies revealed that the enhanced CNS tropism of AAV-PHP.B and AAV-PHP.eB in the C57BL/6J mice might be due to their interaction with the LY6A receptor, which is a GPI-anchored protein that is highly expressed by brain microvascular endothelial cells (Hordeaux et al., 2019; Mathiesen et al., 2020). Interestingly, there is no LY6A homolog in primates thus limiting the utility of these viruses in primates, and further highlighting the need for molecular target identification and validation studies in NHPs as a critical aspect of early research. We have begun to extend this directed evolution strategy to develop novel AAVs that can be used to target primate cells. Multiplexed-CREATE (M-CREATE) implements internal controls to reduce sequencing bias and increase the number of variants identified with enhanced CNS tropism (Kumar et al., 2020). This approach can be iterated across species, testing thousands of candidates in mice to identify top-performing capsids for primate testing. Recently, our lab used M-CREATE to identify systemic variants with enhanced CNS and PNS transduction in both Old World and New World monkeys. In marmosets, we found two variants evolved from AAV-PHP.eB, AAV-CAP-B10 and AAV-CAP-B22, to have enhanced CNS transduction after IV delivery in adult marmosets (Goertsen et al., 2022). AAV-CAP-B10 and AAV-CAP-B22 displayed four and two-fold increased neuronal transduction over AAV9, respectively. Furthermore, AAV-CAP-B10 showed decreased expression in the liver, where expression is typically associated with toxicity, as compared to AAV9. Importantly, broad and robust transgene expression was seen across cortical, subcortical, and cerebellar regions as well as in the dorsal root ganglia (DRG) and the spinal cord (SC).

More recently, we have developed AAV-MaCPNS1 and AAV-MaCPNS2 (Chen et al., 2022). Although these AAVs were designed to target the PNS in rodents, we found that they transduce PNS and CNS in both marmosets and rhesus macaques. Specifically, in adult marmosets, IV delivery of AAV-MaCPNS1/2 capsids carrying fluorescent reporter proteins (i.e., ssAAV:CAG-eGFP or ssAAV:CAG-tdTomato) targeted PNS and CNS more efficiently than AAV9. In the PNS, enhanced transduction was observed in DRG, the small intestine (SI), and the ascending fiber tracts in the dorsal column of the spinal cord (SC). Surprisingly, in the CNS, diffuse brain-wide transduction was seen in regions including the cortex, thalamus, globus pallidus, cerebellum, and brainstem. This tropism was recapitulated in infant rhesus macaques using IV delivery of AAV-MaCPNS1/2. In the PNS, enhanced transduction was seen in the SC, DRG, and gastrointestinal (GI) tract, including the esophagus, colon, and SI. Similar to what was observed in marmosets, in the rhesus monkey CNS, AAV-MaCPNS1/2 capsids mediated enhanced brain-wide transduction, including areas of the cortex, hippocampus, putamen, and brainstem. Additionally, AAV-MaCPNS1/2 displayed an increase in astrocyte transduction over AAV9 in the cortex and thalamus. However, of the two, MaCPNS2 had the greater fold increase in astrocytes. These results demonstrate the potential of these capsids in interrogating peripheral to brain circuitry.

**Fig. 2:**
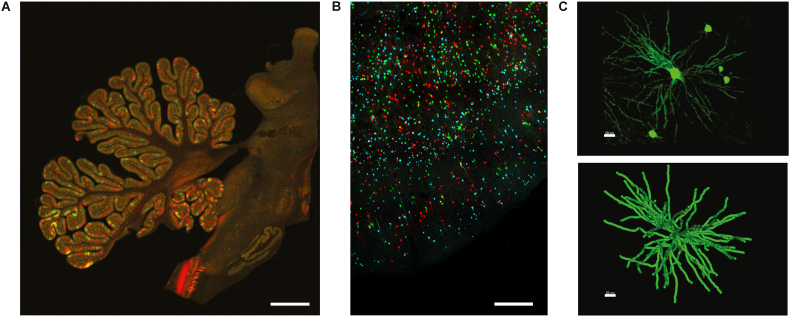
(A) Representative image of MaCPNS1-mediated expression of eGFP and MaCPNS2-mediated expression of tdTomato in the cerebellum and brainstem of an infant rhesus macaque. (B) Multicolor labeling of neurons in cortex after IV administration of a cocktail of 3 fluorescent proteins (ssCAG-mNeonGreen, ssCAG-mRuby2, and ssCAG-mTurquoise2) packaged in AAV-CAP-Mac and (C) morphological reconstruction of a striatal medium spiny neuron in rhesus macaque (scale, 20 uM). Scale bars for A and B = 100 uM.

In addition to M-CREATE, a multi species screening and characterization strategy was used to identify another capsid variant, AAV-CAP-Mac, for systemic brain-wide delivery in Old World Monkeys, including rhesus macaques and green monkeys (*Chlorocebus sabaeus*) (Chuapoco et al., in press). To characterize the transduction properties of AAV-CAP-Mac, pools of AAV-capsids, including AAV9, AAV-PHP.eB, AAV.CAP-B10 and other previously engineered AAVs, were simultaneously injected intravenously into infant rhesus macaques. Each capsid variant contained a single stranded human FXN (frataxin) transgene fused to a hemagglutinin (HA) epitope tag under control of a CAG promoter (ssCAG-hFXN-HA). AAV-CAP-Mac was found to primarily transduce primate neurons in all lobes of the cortex, cerebellum, and multiple subcortical regions. IV injection of AAV-CAP-Mac outperformed its parent capsid AAV9 as well as other previously engineered AAVs, including AAV-PHP.eB. Interestingly, AAV-CAP-Mac also outperformed AAV-CAP-B10, suggesting genetic differences in the BBB even between Old World and New World monkeys. AAV-CAP-Mac, was also shown to have reduced tropism towards the liver across primate species. Further validating the utility of AAV-CAP-Mac for NHP research, delivering a mixture of fluorescent proteins achieved a sparse Golgi stain-like expression pattern that could facilitate large-scale studies of neuronal morphology. Again, in this experiment, AAV-CAP-Mac was seen to target neurons in the cortex and multiple subcortical regions including hippocampus, putamen, thalamus, and caudate, enabling reconstruction of both medium spiny neurons and cortical pyramidal cells (Fig. 2). Additional characterization was also performed in 8-month-old green monkeys. Injections of AAV-CAP-Mac (packaging ssCAG-eGFP) via IV delivery showed broad and strong neuronal expression in all lobes of the cortex and in various subcortical regions. Consistent with results from the rhesus experiments, AAV-CAP-Mac transduced a higher percentage of neurons than AAV9 in sampled cortex and subcortical regions. In summary, these novel AAV variants with tailored properties and tropism, offer greater CNS and/or PNS transduction than AAV9 in NHP.

At present, *in vivo* characterization of AAV-CAP-Mac and AAV-MaCPNS1/2 has been limited to infant rhesus macaques (Chuapoco et al., in press). Higher vector doses could lead to increased immunogenicity due to preexisting neutralizing antibodies, thus, further characterization of these capsids in adult animals is necessary and currently underway. Encouragingly, though, these variants show significantly lower transduction of the liver compared to AAV9, potentially minimizing the risk of hepatotoxicity resulting from high systemic doses. Additionally, while these variants were engineered for systemic delivery, they may also be effective for local delivery. *In vitro* data from human-derived iPSCs supports the increased effectiveness of these AAVs over AAV9 (Chuapoco et al., in press), but further work is needed to test this, as well as the functional efficacy of the genetic cargo delivered by these novel AAVs.

In addition to our efforts, several other groups have also engineered AAVs with unique features that are relevant to NHPs. Using an in-vivo directed evolution strategy, Dalkara et al. engineered a novel AAV variant, 7m8, that specifically transduces photoreceptors in the NHP retina via intravitreal injections (Dalkara et al., 2013). Through iterative refinement, researchers have developed newly engineered variants that induce higher expression in foveal cones than 7m8 in cynomolgus macaques (Byrne et al., 2020). The in-vivo strategy DELIVER (directed evolution of AAV capsids leveraging *in vivo* expression of transgene RNA), has been used to identify muscle-tropic capsids that can be systemically administered to transduce primate muscles with high efficiency in cynomolgus macaques (Tabebordbar et al., 2021). Similarly, rAAV2-retro, which was developed for delivery of genetic cargo to retrogradely targeted cells, has also been tested for use in the primate brain (Bohlen et al., 2020) though see: (Cushnie et al., 2020)).

## 4 AAV-based approaches for targeting specific cell-types in NHPs

Developing tools to target specific cell-types to study their role in normal and disease circuitry remains a major challenge for primate neuroscience. Rodent models often rely on genetically-engineered Cre lines to achieve cell-type specificity. Unfortunately, primate gestational and maturational timelines preclude the widespread use of these genetic engineering approaches in primates (though see: (Drummer et al., 2021; Park et al., 2016; Sasaki et al., 2009; Tomioka et al., 2017)) so a different approach is needed. It is unlikely that capsid engineering alone will achieve the level of cell-type specificity required for NHP neuroscience research. This is due in large part because profiling AAV capsid variants generated by selection experiments is time-consuming and labor-intensive, and most remain uncharacterized (Zolotukhin and Vandenberghe, 2022). Although new molecular and computational tools, such as machine learning, might facilitate capsid profiling, these approaches also have limitations (Zolotukhin and Vandenberghe, 2022). Additionally, studies in NHPs suggest that novel transduction properties may not only arise from unique capsid binding properties, but also from uncharacterized capsid-promoter interactions (Bohlen et al., 2020). Therefore, it is likely that achieving cell-type specificity in NHPs will depend on a combination of BBB-crossing AAV capsid variants and regulatory elements.

Cis-acting regulatory DNA elements, such as promoters and enhancers, are sequences of DNA that proteins bind to in order to initiate and increase the likelihood of transcription respectively (Levine, 2010; Wittkopp and Kalay, 2012). Thus promoters and enhancers can determine the level of transgene expression and the cells they are expressed in. Ubiquitous promoters, such as cytomegalovirus (CMV), chicken β-actin (CBA), human elongation factor 1 alpha (EF1α) or combinations of these such as CMV early enhancer/chicken beta actin (CAG), drive high levels of transgene expression in most cell-types (Haery et al., 2019). However, high, widespread transgene expression is not always desired and can evoke immune responses to the transgene product (Perez et al., 2020; Samelson-Jones et al., 2020). Alternatively, cell-type specific promoters can be incorporated into the AAV cargo. These can be used, for instance, to target neurons (synapsin 1) or astrocytes (glial fibrillary acidic protein), or even more specifically dopaminergic neurons, cerebellar Purkinje cells, or parvalbumin (PVALB) neurons in the brain (El-Shamayleh et al., 2017; Hoshino et al., 2021; Matsuzaki et al., 2014; Nitta et al., 2017; Shinohara et al., 2016; Stauffer et al., 2016). Promoter sizes can range anywhere from ∼100 bp to 1000 bp (Domenger and Grimm, 2019). Due to the AAV's size limitation, ongoing efforts are focused on identifying shorter, and phylogenetically conserved, regulatory element sequences to direct cell-type specific transgene expression across species (de Leeuw et al., 2016; Domenger and Grimm, 2019; Matsuzaki et al., 2014; Nathanson, 2009).

Recently, chromatin profiling techniques coupled with next-generation sequencing led to the discovery of putative enhancers that are less than 600 bp and can drive cell-type specific activation of genes (Buenrostro et al., 2013; Cusanovich et al., 2015; Fang et al., 2021; Grandi et al., 2022; Graybuck et al., 2021; Hrvatin et al., 2019; Mich et al., 2021; Nair et al., 2020; Preissl et al., 2018; Rubin et al., 2020; Visel et al., 2013; Vormstein-Schneider et al., 2020). A distal-less homeobox (*Dlx*) gene enhancer sequence that targets GABAergic interneurons in the telencephalon of several vertebrate species including mouse and marmoset was identified (Dimidschstein et al., 2016; A. T. Lee et al., 2014; Zerucha et al., 2000). Additionally, the mouse ortholog of the *Dlx5/6* enhancer (mDLX5/6), which is only ∼400 bp, packaged into either AAV1 or AAV9, showed similar specificity for GABAergic interneurons when locally injected into area V1 of the primary visual cortex of rhesus macaques (De et al., 2020). Mich et al. further optimized the human ortholog of the *Dlx5/6* enhancer (hDLXI5/6i) by engineering a triple tandem of core elements taken from hDLXI5/6i and called it hDLX2.0 (Mich et al., 2021). AAV-PHP.eB containing hDLX2.0 upstream of a minimal beta-globin promoter and super yellow fluorescent protein-2 (SYFP2) reporter transduced GABAergic interneurons in *ex vivo Macaca nemestrina* cortical slice cultures and human neocortical slice cultures (Mich et al., 2021). Putative enhancers for targeting PVALB-expressing interneurons have similarly been identified, packaged in AAV-PHP.eB and tested in mice via retro-orbital injections, and in marmoset and macaque via local or intraparenchymal injections (Lawler et al., 2022; Mich et al., 2021; Vormstein-Schneider et al., 2020). These enhancers either targeted PVALB interneurons broadly or specific sub-classes of PVALB interneurons in the neocortex in both mouse and NHP. To identify regulatory elements that can drive faithful expression across species using AAV vectors, the selection method has largely focused on sequences that are conserved across species. However, Mich et al. reported that certain PVALB enhancer sequences present in the open chromatin analyses of the human neocortex but not in the mouse neocortex, were still able to drive selective expression in PVALB neurons in the mouse brain (Mich et al., 2021; Vormstein-Schneider et al., 2020). Thus, to minimize the number of experimental animals used for *in vivo* screening, it may be advantageous to develop machine-learning classifiers that can identify DNA sequence patterns important for driving species-agnostic cell-type specific activation (Lawler et al., 2022).

Single-cell and single-nucleus transcriptomics studies of the rodent and primate brain have revealed the molecular complexity and diversity of cell-types present based on their gene expression profiles (Hodge et al., 2019; Tasic et al., 2016, 2018; Zeisel et al., 2015). In the primary motor cortex alone, there are potentially 45 conserved cell-types among mouse, marmoset and human (Callaway et al., 2021). Only once a cell-type has been molecularly defined can researchers begin to identify DNA regulatory elements that are required for cell-type specific gene activation, and guide the development of tailored targeting strategies for treatment and functional interrogation. We have shown that broad CNS transduction in NHP is possible using the ubiquitous CAG promoter in our recently engineered vectors (AAV-CAP-Mac, AAV-MaCPNS1 and AAV-MaCPNS2) (Chen et al., 2022; Chuapoco et al., in press). These variants, which show comparatively higher neuronal transduction than AAV9 in NHPs, can be used to screen regulatory elements that specifically target neuronal subpopulations. One caveat of this approach is that injecting multiple AAVs with different enhancer and promoter elements in the same animal may cause interference between the regulatory elements, resulting in a loss of specificity compared to independent delivery (Mehta et al., 2019; Pouchelon et al., 2022). This may confound interpretation of pooled screens of putative regulatory elements.

Further cell- and tissue-type specificity can be achieved by incorporating microRNA (miRNA) target site sequences (∼22 nucleotides) into the 3’ untranslated coding region of AAVs. These bind to complementary miRNA sequences wherever they are expressed and inhibit mRNA expression. This approach has been used to detarget transgene expression from primary sensory neurons (miR183) and liver (miR122) to reduce dose-dependent toxicity in NHPs (Hordeaux et al., 2020; Kochunov et al., 2015). Recently, a database of miRNA expression across 196 primary cell-types was generated, enabling the potential testing of numerous combinations of miRNA-binding transgene cassettes (Patil et al., 2022). With spatial transcriptomics we can characterize the cell type-specific expression of various combinations of systemically-delivered AAV capsids and cargo, with the goal of expanding the gene delivery toolkit for NHPs (Jang et al., 2023).

## 5 Future directions

The effective delivery of sensors, effectors, and reporters for circuit tracing and manipulation, largely depends on the vector used. However, differences in brain size and immune function have hampered the widespread adoption of genetic technology in monkeys. Thus, the engineering of more efficient and specific viral vectors to target the CNS in NHPs addresses many of the challenges that have inhibited progress in translating rodent disease biology to better therapies and therapeutic approaches. Here, we review newly engineered systemic capsid variants that address these challenges. Specifically, using an adapted, cross-species directed evolution approach, our group has identified new variants that can transduce neuronal cells in CNS and PNS via peripheral injection in multiple NHP species commonly used in research.

In marmosets, AAV-CAP-B10 and AAV-CAP-B22, variants of AAV-PHP.eB, were identified to have enhanced CNS transduction compared to AAV9. In both marmosets and rhesus macaques, AAV-MaCPNS1 and AAV-MaCPNS2 variants transduced cells in both CNS and PNS. This may be particularly useful for studies where widespread transgene expression is desired as infusions in NHPs typically require multiple sites of injection to cover whole areas of tissue; however, this remains to be tested in relation to distributed brain function and/or behavior. In both rhesus macaques and green monkeys, AAV-CAP-Mac was found to transduce a higher percentage of neurons than AAV9. Importantly, these variants show significantly lower transduction in the liver compared to AAV9, thereby minimizing the risk of hepatotoxicity.

Still, while these novel AAVs may provide a new and necessary tool for delivering genetic cargo into the primate brain, many technical issues still remain. Below we briefly discuss these issues.

1. *Achieving cell-type specificity in NHPs.* For decades, studies in NHPs have relied on lesions, reversible inactivation, and electrophysiology to elucidate the role of specific regions in a particular function (Balan et al., 2019; Dal Monte et al., 2015; Lak et al., 2014; Rudebeck et al., 2013). While this has provided invaluable insight into distributed circuits that underlie behavior, these studies are limited by their cell-type agnostic nature. Lesioning and reversible inactivation generally impact all cells in a particular region. In addition, lesion studies have resulted in conflicting reports on observed behaviors within the same region of the brain, often due to unintended damage to fibers of passage (Rudebeck et al., 2013). Similarly, electrophysiological recording techniques cannot differentiate molecular cell-types, and rely on electrophysiological-specific characterization (e.g. early-firing, late-firing, ramping, etc.). For example, in the ventral tegmental area, a minority of neurons share the same electrophysiological properties as dopamine neurons, leading to questions on whether some recordings have been misattributed to dopamine (Ungless and Grace, 2012). To address this issue, rodent studies have used TH-Cre mice to target dopamine-expressing VTA neurons for the expression of sensors and effectors (Bariselli et al., 2016; Lindeberg et al., 2004). However, in NHPs, Cre-lines do not yet exist or are not widely used. It is unlikely that capsid engineering alone will enable cell-type specificity. Instead, it is likely that a combination of capsid and DNA regulatory elements will achieve cell-type specificity. To this end, AAV-Cap-Mac and AAV-MaCPNS1/2 can be used to screen regulatory elements that specifically target neuronal subpopulations.
2. *Effective delivery and functional efficacy of genetic cargo.* Advances in genetic cargo, like opsins and DREADDs, have been critical in dissecting circuits that are thought to contribute to disease -- in rodents. However, these techniques have not been widely adopted in NHPs because of difficulties in delivering genetic cargo efficiently into the primate brain. Currently, only a few published studies in NHPs have demonstrated successful delivery of opsins, with AAV5 and AAV9 being amongst the most commonly used vectors (Tremblay et al., 2020). Moreover, outcome measures-anatomy, physiology, and behavior in these studies-vary greatly (Bliss-Moreau et al., 2022). For example, AAV5 has been shown to preferentially target some brain regions but not others (Roseboom et al., 2021). Still, a major hurdle remains in determining the extent in which systemic delivery can express effector-cargo in a sufficient proportion of cells to affect behavior. Even so, infecting a small proportion of cells can still help us better understand the contributions of a small number of cells on behaviors as the functional efficacy of sensor-cargo does not necessitate a large proportion of cells. Currently, the effective delivery and functional relevance of sensors and effectors have not been tested using AAV-CAP-Mac or AAV-MaCPNS1/2. Further work is needed to show the functional efficacy of the genetic cargo delivered by these novel AAVs.

Understanding the emergence or origins of brain-based disease requires coordinated cross-species research. To this end, NHP models are particularly important because of their shared biology with humans. However, tools for interrogating anatomical pathways and functional circuits will need to be translated for widespread use in NHPs. While the vectors presented here address many common technical challenges seen in NHPs, there is a continued need for more efficient and specific AAVs. We hope that further optimization of these vectors can lead to more efficient delivery and ultimately, lead to new tools for the study of the primate brain and the development of new treatments for brain-based disorders.

## CRediT authorship contribution statement

**Lillian J. Campos:** Writing – original draft, Writing – review & editing. **Cynthia M. Arokiaraj:** Writing – original draft, Writing – review & editing. **Miguel R. Chuapoco:** Writing – review & editing. **Xinhong Chen:** Writing – review & editing. **Nick Goeden:** Writing – review & editing. **Viviana Gradinaru:** Writing – original draft, Writing – review & editing. **Andrew S. Fox:** Writing – original draft, Writing – review & editing.

## Declaration of competing interest

The authors declare the following financial interests/personal relationships which may be considered as potential competing interests: Viviana Gradinaru reports a relationship with Capsida Biotherapeutics that includes: board membership. Nick Goeden reports a relationship with Capsida Biotherapeutics that includes: employment.

## Data Availability

No data was used for the research described in the article.
