# Supplementary material for: Advances in AAV technology for delivering genetically encoded cargo to the nonhuman primate nervous system
Source: Curr Res Neurobiol. 2023 Apr 7;4:100086. doi: 10.1016/j.crneur.2023.100086 (PMC10313870; doi:10.1016/j.crneur.2023.100086)
Supplement: Multimedia component 1 [file mmc1.pdf]

Peer Review Overview

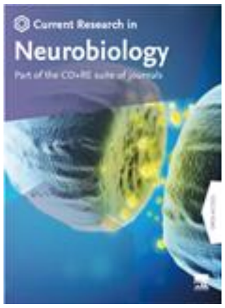

Manuscript Title: Advances in AAV Technology for Delivering Genetically Encoded Cargo to the Nonhuman Primate Nervous System

Received	Sep 30, 2022
1st Decision	Nov 05, 2022
1st Revision Submitted	Feb 05, 2023
Accepted	Mar 17, 2023

1st Decision letter

Reference: CRNEUR-D-22-00128
Title: Advances in AAV Technology for Delivering Genetically Encoded Cargo to the Nonhuman Primate Nervous System
Journal: Current Research in Neurobiology

Dear Dr. Campos,

Thank you for submitting your manuscript to the Special Issue in Current Research in Neurobiology: Illuminating the Monkey Brain: Organization, Networks and Circuits.

The reviews of your manuscript are appended to this email. Both reviewers were generally positive but both have made several suggestions that would substantially improve the quality of the manuscript.

I invite you to resubmit your manuscript after addressing these comments and suggestions. Please resubmit your revised manuscript by Jan 04, 2023.

When revising your manuscript, please consider all issues mentioned in the reviewers' comments carefully: please outline every change made in response to their comments and provide suitable rebuttals for any comments not addressed. Please note that your revised submission may need to be re-reviewed.

Current Research in Neurobiology values your contribution and I look forward to receiving your revised manuscript.

Kind regards,

Yogita Chudasama
Associate Editor
Current Research in Neurobiology

Comments from Editors and Reviewers:

Reviewer #1:

Summary:
This is a very well written review manuscript by Campos and colleagues entitled, "Advances in AAV technology for delivering genetically encoded cargo to the nonhuman primate nervous system." The review broadly discusses AAV capsid and genome engineering advances that are underway and their relevance and utility in the non-human primate model. I have a few minor comments for the authors to consider, but otherwise, I think this is a superb review.

Section: Advantages of AAVs for circuit interrogation and gene delivery

Paragraph 2

Sentence 1 - I would argue that the immune response is another critical and underappreciated component.

Sentence 3 to the end - While I understand that the author's backgrounds and interests are in support of intravenous delivery, I would argue that are many advantages to direct intraparenchymal injections over IV that are being ignored. For example, while obviously invasive, direct intraparenchymal injections are not that hard to do with proper training. With intraparenchymal delivery, one can achieve both dense and robust local transduction, and with the right capsid, diffuse projection labeling that is far greater than anything reported with IV delivery. Finally, a simple resolution to being required to make multiple intraparenchymal injections, would be to develop vectors that more effectively diffuse through the parenchyma. Finally, intraparenchymal delivery avoids the hepatotoxicity and DRG toxicity. Again, my point being, if the authors are going to show one side of the coin, it is only fair that they are also honest about the benefits of the other side.

Section: Capsid engineering for systemic delivery to achieve tissue specific biodistribution in NHPs

Paragraph 2
I have a problem with using the term "robust" to describe both transduction in primates in general, but specifically here to describe transduction of AAV-MaCPNS1/2. While I agree with the authors that the labeling is better than AAV9, I would argue that neither capsids is robust given that individual neurons can be observed across histological sections. For example, in figure 2A, the labeling is from individual Purkinje cells in the cerebellum, I can make out Purkinje cell bodies in the Purkinje cell layer, and I can see individual Purkinje cell dendritic fields in the molecular layer. A better term may be, "diffuse," to describe the observed labeling, and that the labeling is greater compared to AAV9. I wish to also be clear, I believe in the importance of this work, and the development of better capsids, but I believe there is a tremendous amount of work yet to be done.

Section: Discussion

Paragraph 1: "This is particularly useful for studies where widespread transgene expression is desired as infusions in NHPs typically require multiple sites of injection to cover whole areas of tissue."
This is a very broad statement that is going to be received with a lot of skepticism from primate

neuroscientific researchers who have been burned trying to do opto- or chemo-genetic experiments and have failed. As I pushed on above, these intravenously delivered capsids certainly gives diffuse (or widespread) transduction. However, without any sort of physiological or behavioral proof, I do not suggest the authors argue that intravenous delivery provides dense enough labeling in any one structure to have functional relevance. This skepticism comes from lots of experiments where primate labs have attempted to intraparenchymally deliver viral injections - which gives greater labeling within a structure - and were still unable to evoke some sort of behavioral responses; See Tremblay et al (2020) - An Open Resource for Non-Human Primate Optogenetics, Bliss-Moreau et al (2022) - A Pragmatic Reevaluation of the Efficiency of Nonhuman Primate Optogenetics for substantiating evidence, and Daw et al (2022) - Direct comparison of epifluorescence and immunostaining for assessing viral mediated gene expression in the primate brain.

Paragraph 3: "Moreover, outcome measure- anatomy, physiology, and behavior in these studies vary greatly"
Please cite: Bliss-Moreau et al (2022) - A Pragmatic Reevaluation of the Efficiency of Nonhuman Primate Optogenetics for substantiating evidence

Reviewer #2:

Although the topic of this review is undoubtedly very important, the paper does not provide the reader with a broader understating of the viral vectors and its use in NHPs. It also fails to provide specific knowledge about the AAV. The chaotic organization of the information and lack of fluid transition between the paragraphs makes it hard to read. Furthermore, after reading the paper, it is not clear weather the authors aimed to review current state of the AAVs in NHP, the importance of designing new AAV serotypes specific for NHP, or just give the quick overview on the three artificially engineered AAV. The article would have more impact if more attention were paid to the organization and the definition of terms and clarity. Experts dont need to read reviews - novices or people who want to learn about a field do. The article should be pitched to that audience.

Specific comments:

Graphical abstract:

It is really pretty, and well describes the artificial AAV capsids.

I might be wrong, but it looks to me like it has been done in Biorender, however there is no reference to it (and I believe the company requires you to provide the citation).

Although it describes well those three artificial AAVs, it does not seem to be connected to the papers' abstract, and somehow it is disconnected from the main text.

Nonhuman primate as a valuable model for study of human diseases

First paragraph:

Vague and nonspecific example: "For example, rodent studies using optical stimulation and imaging techniques have implicated distinct cell types and projections underlying specific biological function that likely contribute to many brain-based disorders"

What optical stimulation?, what cell types?, what function? what disorder?

This example through its vagueness looses its purpose of being an example, because the reader still does not have an idea of what those techniques can accomplish

Missing citation after "However, nonhuman primates (NHPs) are well-suited to bridge this gap as their recent evolutionary divergence from a common ancestor have endowed them with may anatomical, physiological, and etiological similarities to humans"

2nd paragraph

"Furthermore, proof-of-principle for modeling aspects of prevalent neurological diseases like Alzheimer's in NHPs has been achieved through direct injection of wildtype serotypes" followed by "However, more efficient and widespread distribution of vectors, as well as the translation of the modern toolkit to NHPs will be critical (…)

What wild type serotypes? Serotypes of what? What is a serotype?

What serotypes are currently used in rodents? In NHP?

What is wrong with these serotype? Why aren't they efficient?

This paragraph aims to provide a rationale why do we need NHP models of human diseases, and why we need better AAVs to achieve better models of human diseases… however it never really provides any arguments that the current models are not sufficient

3rd paragraph

This paragraph gives an overview of different species of monkey used in research. It seems very disconnected from the narrative. It also makes statements like e.g. "Old world monkeys are useful models of cognition, attention, and memory, among other." But the authors never say why are they good models?

4th paragraph

This paragraph describes the differences between the rodent and NHP brain physiology. Although this is a good paragraph to have in section dedicated to describing why NHP are valuable model of study human diseases, it is hard to see how it relates to the rest of the section. Furthermore, the authors focus on specific to NHP neuronal cells, however, the focus of this review are not AAV that target specific cells, but rather AAVs that are capable of infecting all brain

Overall this section needs major revisions. The paragraphs seem disconnected, and the message is somehow scattered. It needs better organization. After reading this paragraph, the reader still has hard time to give specific examples of why NHP are good models for human disease

Advantages of AAVs for circuit interrogation and gene delivery

First paragraph needs citations. The authors throw a lot of information with no citations…. This is not acceptable / scholarly

Although this section is titled "Advantages of AAVs for circuit interrogation and gene delivery" the authors only mention one AAV serotype, never talk about other viral vectors, and why would AAV be better than others…

Inconsistency among different parts of the paper. E.g. this section starts with "The ability to define, monitor, and manipulate a neural circuit requires precise delivery of reporters… (…)" the AAVs mentioned in the graphical abstract do not address the problem of specificity, but rather provide brain-wide expression

"Additionally, AAVs are capable of transducing both dividing and non-dividing cells with stable, long-term transgene expression in post-mitotic cells"

Why is that important?

Citation?

How does is compare to other viral vectors?

Why is that important for NEURO?

"For the study and treatment of neurological and neurodegenerative diseases such as Parkinson-s disease, widespread transgene expression within the CNS is desired"

Citation?

Why is it important to have a widespread transgene expression?

Studying how?

How using the AAV to study PD? To model the disease?

I would argue that for treatment we need specificity not widespread expression within the CNS. We have widespread effects of the systemic drugs e.g. LDopa, and the WIDESPREAD nature of its action is in fact problematic. Focused, specific treatment is much more desired

"Thus, systemically-delivered AAV variants that can transduce neurons with higher efficacy are still needed for NHP studies"

The authors only brought up the AAV9… there are multiple other serotypes that transduce neurons

This section is titled "Advantages of AAVs for circuit interrogation and gene delivery", however I do not think that the content follows. There is only one sentence that might provide some exemplary characteristic of AAVs that give them advantages over OTHER viral vectors, however the authors have never even mentioned other viral vectors

What are other viral vectors?

Why AAVs are better suited for NHP studies than e.g. lenti virus?

What characteristics of viral vectors are desired for NHP studies?

What are serotypes?

What AAV serotypes are there?

Why AAVs are better for circuit interrogation than other viral vectors?

Capsid engineering for systemic delivery to achieve tissue specific biodistribution in NHPs

This section is practically the only one that is related to the graphical abstract. Although it does describe the artificially engineered capsids, it fails to provide crucial information to the reader e.g.:

How does capsid and capsid modification relate to tropism?

What is the capsid? What does it do?

Why does the capsid matter for the viral vectors?

How do capsids and serotypes relate?

How do capsid affect tropism and efficacy?

What types of capsids are there already?

Cell-type specific targeting

rAAV have not been only tested in NHP, but have been used in variety of studies involving optogenetics, neuroanatomical labeling…

it is not clear how capsid design relate to cell-specificity… in the example of the rAAVs it is not per se the capsid design as the injection site that specifies the cell type that is transduced… yes the retro nature of this viral capsid allows for the retrograded uptake, but the cell specificity is not achieved in this manner… e.g. if you inject rAAV to SC you will express the genes carried by this viral vector in all (except GABAergic cells which for unknown reason do not work with rAAVs) projections to the SC… so there is not cell specificity

Why are promoters and enhancers important?

What do enhancers and promoters do?

How do enhancers and promoters could achieve cell-specificity?

"Single-cell and single nucleus transcriptomics studies of the rodent and primate brain have revealed the molecular complexity and diversity of cell types present". How do single-cell and single-nucleus transcriptomics and the molecular complexity that they reveal, relate to the cell-specificity of the AAV?

How do these studies can help creating new enhancers and promoters?

"Importantly, these enhancers tend to be smaller in size in comparison to promoters, allowing for more flexibility with AAV payload which is limited to 4.7kb"

Lack of citation

Why is the payload important?

What is the difference between enhancers and promoters?

Why is small size of the enhancers important?

"AAV-PHP.eB containing hDLX2.0 upstream of minimal beta-globin promoter and super yellow fluorescent protein-2 (SYFP2) reporter reportedly transduced GABAergic interneurons ex vivo (…)"

Lack of citation

"Combinatorial methods involving dual vector injections with distinct enhancers and promoter elements have also been attempted to target certain cell-types"

what are these "combinatorial methods"?

Not clear what is this method and how it relates to cell specificity…

The title of this section implies that the authors will discuss variety of cell types within the brain and how they can be targeted using specific AAV serotypes and/or enhancers and promoters. However, the authors never mentioned all the classes and subclasses of the neurons that are there… what cells are conserved between species and what are not? and what are the ones that are specific for NHP? (they only give few examples)

Do the enhancers that work for the different classes in rodents work the same in NHP?

Discussion

The discussion focuses on summarizing the few scatter information about the three artificially engineered AAVs. As a result it seems disconnected from the whole narrative.

1st Author Response Letter

Response to comments from Editors and Reviewers:

Comments from Reviewer 1:

<u>*Summary:*</u>

This is a very well written review manuscript by Campos and colleagues entitled, "Advances in AAV technology for delivering genetically encoded cargo to the nonhuman primate nervous system." The review broadly discusses AAV capsid and genome engineering advances that are underway and their relevance and utility in the non-human primate model. I have a few minor comments for the authors to consider, but otherwise, I think this is a superb review.

Section: Advantages of AAVs for circuit interrogation and gene delivery

Paragraph 2

R1Comment1: *Sentence 1 - I would argue that the immune response is another critical and underappreciated component.*

We agree with the reviewer's comment. We have edited the manuscript to include immune responses as a key component of AAV's safety, efficacy and tropism. Paragraph 3, Sentence 5 now reads:

> **"**The route of AAV administration, dose, age at the time of injection, and preexisting neutralizing antibodies against it in the host, are all key determinants of an AAV's safety, efficacy and tropism"

R1Comment2: *Sentence 3 to the end - While I understand that the author's backgrounds and interests are in support of intravenous delivery, I would argue that are many advantages to direct intraparenchymal injections over IV that are being ignored. For example, while obviously invasive, direct intraparenchymal injections are not that hard to do with proper training. With intraparenchymal delivery, one can achieve both dense and robust local transduction, and with the right capsid, diffuse projection labeling that is far greater than anything reported with IV delivery. Finally, a simple resolution to being required to make multiple intraparenchymal injections, would be to develop vectors that more effectively diffuse through the parenchyma. Finally, intraparenchymal delivery avoids the hepatotoxicity and DRG toxicity. Again, my point being, if the authors are going to show one side of the coin, it is only fair that they are also honest about the benefits of the other side.*

We have included intraparenchymal injections as a method of delivery to the brain. Our manuscript now reflects this change. Paragraph 3, Sentence 7-11 now reads:

> **"**There are many advantages to direct in-brain injection. In fact, most studies to date have directly injected viruses into the brain to deliver genetic cargo to specific regions. This has been performed in animal models of PD to target the putamen or substantia nigra (Bartus et al, 2013 , Kells et al, 2012, Muramatsu et al, 2010, Christine et al, 2009, Kaplitt et al, 2007). Despite its many advantages, which include relatively dense and robust expression surrounding the infusion site, targeting large, diffuse, or spatially distributed regions can require multiple injections. Thus, this route of administration is most suitable for localized targets. Covering entire brain regions

> remains a challenge due to the size of the primate brain and often requires multiple craniotomies (Wang, 2021,). Systemic delivery of AAVs obviates the need for multiple direct injections and importantly reduces the health risks associated with extremely long and highly invasive surgeries. As a therapeutic approach, systemic administration via a single injection might be a safer alternative toward achieving brain wide gene transduction (Kimura and Harashima, 2022, Bourdenx et al, 2014). Ultimately, increased efficacy of BBB-crrossing AAVs may be combined with other technologies to achieve localized expression, as we suggest below."

Section: Capsid engineering for systemic delivery to achieve tissue specific biodistribution in NHPs

Paragraph 2

R1Comment3: *I have a problem with using the term "robust" to describe both transduction in primates in general, but specifically here to describe transduction of AAV-MaCPNS1/2. While I agree with the authors that the labeling is better than AAV9, I would argue that neither capsids is robust given that individual neurons can be observed across histological sections. For example, in figure 2A, the labeling is from individual Purkinje cells in the cerebellum, I can make out Purkinje cell bodies in the Purkinje cell layer, and I can see individual Purkinje cell dendritic fields in the molecular layer. A better term may be, "diffuse," to describe the observed labeling, and that the labeling is greater compared to AAV9. I wish to also be clear, I believe in the importance of this work, and the development of better capsids, but I believe there is a tremendous amount of work yet to be done.*

We agree. We have changed the term "robust" to the suggested term "diffuse". Paragraph 2, Sentence 4 now reads:

> **"**Although these AAVs were designed to target the peripheral nervous system in rodents, we found that they transduce PNS and CNS in both marmosets and rhesus macaques. Specifically, in adult marmosets, IV delivery of AAV-MaCPNS1/2 capsids carrying fluorescent reporter proteins (i.e., ssAAV:CAG-eGFP or ssAAV:CAG-tdTomato) were found to target PNS and CNS more efficiently than AAV9. In the PNS, enhanced transduction was observed in DRG, the small intestine (SI), and the ascending fiber tracts in the dorsal column of the spinal cord (SC). Surprisingly, in the CNS, diffuse brain-wide transduction was seen in regions including the cortex, thalamus, globus pallidus, cerebellum, and brainstem."

Section: Discussion

R1Comment4: *Paragraph 1: "This is particularly useful for studies where widespread transgene expression is desired as infusions in NHPs typically require multiple sites of injection to cover whole areas of tissue."*

This is a very broad statement that is going to be received with a lot of skepticism from primate neuroscientific researchers who have been burned trying to do opto- or chemo-genetic experiments and have failed. As I pushed on above, these intravenously delivered capsids certainly gives diffuse (or widespread) transduction. However, without any sort of physiological or behavioral proof, I do not suggest the authors argue that intravenous delivery provides dense enough labeling in any one structure to have functional relevance. This skepticism comes from lots of experiments where primate labs have attempted to intraparenchymally deliver viral injections - which gives greater labeling within a structure - and were still unable to evoke some sort of behavioral responses; See Tremblay et al (2020) - An Open Resource for

Non-Human Primate Optogenetics, Bliss-Moreau et al (2022) - A Pragmatic Reevaluation of the Efficiency of Nonhuman Primate Optogenetics for substantiating

evidence, and Daw et al (2022) - Direct comparison of epifluorescence and immunostaining for assessing viral mediated gene expression in the primate brain.

We appreciate the reviewer's concern. We have further elaborated in our manuscript that while we believe that these capsids may be helpful for delivery of genetic cargo, this has yet to be validated. Future Directions-Paragraph 2, Sentences 3 now reads:

> "This may be particularly useful for studies where widespread transgene expression is desired as infusions in NHPs typically require multiple sites of injection to cover whole areas of tissue; however this remains to be tested in relation to distributed brain function and/or behavior."

Additional changes were made to Future Directions-Paragraph 6, Sentence 7-9. This now reads:

> "Still, a major hurdle remains in determining the extent in which systemic delivery can express effector-cargo in a sufficient proportion of cells to affect behavior. Even so, infecting a small proportion of cells can still help us better understand the contributions of a small number of cells on behaviors as the functional efficacy of sensor-cargo does not necessitate a large proportion of cells. Currently, the effective delivery and functional relevance of sensors and effectors have not been tested using AAV-CAP-Mac or AAV-MaCPNS1/2. Further work is needed to show the functional efficacy of the genetic cargo delivered by these novel AAVs."

R1Comment5: *Paragraph 3: "Moreover, outcome measure- anatomy, physiology, and behavior in these studies vary greatly"*

Please cite: Bliss-Moreau et al (2022) - A Pragmatic Reevaluation of the Efficiency of Nonhuman Primate Optogenetics for substantiating evidence

We have updated to include the suggested reference Bliss-Moreau et al., 2022.

Comments from Reviewer 2:

Although the topic of this review is undoubtedly very important, the paper does not provide the reader with a broader understating of the viral vectors and its use in NHPs. It also fails to provide specific knowledge about the AAV. The chaotic organization of the information and lack of fluid transition between the paragraphs makes it hard to read. Furthermore, after reading the paper, it is not clear weather the authors aimed to review current state of the AAVs in NHP, the importance of designing new AAV serotypes specific for NHP, or just give the quick overview on the three artificially engineered AAV. The article would have more impact if more attention were paid to the organization and the definition of terms and clarity. Experts dont need to read reviews - novices or people who want to learn about a field do. The article should be pitched to that audience.

Specific comments:

R2Comment1: *Graphical abstract:*

It is really pretty, and well describes the artificial AAV capsids.

I might be wrong, but it looks to me like it has been done in Biorender, however there is no reference to it (and I believe the company requires you to provide the citation).

Our original manuscript has a Biorender citation in the Acknowledgements. We are choosing to keep our citation in the Acknowledgements section of our manuscript, which is in line with BioRender's terms of usage.

R2Comment2: *Although it describes well those three artificial AAVs, it does not seem to be connected to the papers' abstract, and somehow it is disconnected from the main text.*

This is intentional. A number of the other sections are largely reviewing the need for these tools. We believe the field is better suited by a graphical abstract that targets these specific advances, rather than the rationale for why they are important. We hope that this is made clear in the revised manuscript. Additionally, we have made edits to the graphical abstract to include Future Directions which are discussed in the manuscript.

Nonhuman primate as a valuable model for study of human diseases

First paragraph:

R2Comment3: *Vague and nonspecific example: "For example, rodent studies using optical stimulation and imaging techniques have implicated distinct cell types and projections underlying specific biological function that likely contribute to many brain-based disorders"*

What optical stimulation?, what cell types?, what function? what disorder?

This example through its vagueness looses its purpose of being an example, because the reader still does not have an idea of what those techniques can accomplish

Per the reviewer's suggestions, we have further elaborated for clarity. The first paragraph now reads:

"Recent advances in genetic technologies have made it possible to control and image neuronal circuits in living animals, through the delivery of various effectors, sensors, and reporters to the brain (Fenno et al., 2011; Boyden et al., 2005; Wang et al., 2019;Yang & Yuste, 2017). This breakthrough in technology has advanced our understanding of neural circuits, cell-types, molecules, neurotransmitters, and gene regulatory elements that work together to contribute to the progression of disease (e.g.(Fadok et al., 2018;Coley et al., 2021;Cummings and Clem, 2020;Pignatelli and Beyeler, 2019, Xu et al., 2019)). For example, research on anxiety-relevant circuits has leveraged optical control of specific cell-types (e.g. somatostatin and corticotrophin-releasing hormone expressing cells) and their projections to threat-relevant regions (e.g. central amygdala to periaqueductal gray interneurons) in order to elucidate multiple distinct mechanisms that underlie specific aspects of threat responding behavior (Fadok et al., 2017;Holley & Fox, 2022, Ciocchi et al.,2010, Tovote et al., 2016). This work has far-reaching implications for our understanding of anxiety disorders, by identifying multiple distinct mechanisms that likely contribute to differences in symptomatology. Similarly, in basic research studies of the mechanisms

relevant to neurodegenerative diseases like Parkinson's, optical inhibition of cells in the subthalamic nucleus of parkinsonian rodents was sufficient to improve 6-hydroxydopamine-induced forelimb akinesia, opening the door to potential treatment avenues for patients with PD (Yoon et al, 2015)."

R2Comment4: *Missing citation after "However, nonhuman primates (NHPs) are well-suited to bridge this gap as their recent evolutionary divergence from a common ancestor have endowed them with may anatomical, physiological, and etiological similarities to humans"*

We have updated to include the reference to Petrides and Pendaya, 1999,2002; Ongurand Price,2000; Kalin & Shelton, 2003; Phillips et al, 2014

2nd paragraph

R2Comment5: *"Furthermore, proof-of-principle for modeling aspects of prevalent neurological diseases like Alzheimer's in NHPs has been achieved through direct injection of wildtype serotypes" followed by "However, more efficient and widespread distribution of vectors, as well as the translation of the modern toolkit to NHPs will be critical (…)*

We have removed this paragraph for clarification. We have integrated relevant parts in other sections of the manuscript.

R2Comment6: *What wild type serotypes? Serotypes of what? What is a serotype?*

We have elaborated on the definition of a serotype. This change is reflected in section "AAVs enable gene delivery and circuit interrogation", paragraph 3, sentences 1-2:

> "To date, 12 distinct naturally-occurring or *wildtype* serotypes of AAVs, (AAV1-12) have been identified in humans and NHPs (Agbandje-McKenna and Kleinschmidt, 2011). Each of these serotypes differs in capsid structure and, therefore, tropism (Agbandje-McKenna and Kleinschmidt, 2011)."

R2Comment7: *What serotypes are currently used in rodents? In NHP?*

We have included serotypes that are currently used in rodents and NHPs. This change is reflected in section "AAVs enable gene delivery and circuit interrogation", paragraph 3, sentences 1-8:

> "To date, 12 distinct naturally-occuring or *wildtype* serotypes of AAVs, (AAV1-12) have been identified in humans and NHPs (Agbandje-McKenna and Kleinschmidt, 2011). Each of these serotypes differs in capsid structure and, therefore, tropism (Agbandje-McKenna and Kleinschmidt, 2011). The most commonly used serotypes in rodent research are AAV2, AAV5, AAV8, and AAV9, which transduce the CNS, although some transduce other organs as well (Aschauer et al., 2013). In NHPs, the most commonly used serotypes are AAV5 and AAV9 (Trembley et al.,2020). AAV9 has been particularly widely studied because of its ability to cross the BBB and has been employed in several CNS-targeted gene therapies (Y. A. Chan & Deverman, 2022, Song et al, 2022, W. Chen et al., 2021, Yang et al, 2014, Zhang et al, 2011, Foust et al., 2009, Choi et al, 2006). Efforts have also been made to characterize other serotypes that also have the capability to cross the BBB. For example, Gao et al. cloned and identified more than 100 novel rAAVs from human and NHP tissues (Gao et al, 2004, Gao et al, 2002). Among these, AAVrh8, AAVrh10 and AAVhu32 were found to cross the BBB with high efficiencies, similar to AAV9. "

R2Comment8: *What is wrong with these serotype? Why aren't they efficient?*

Different serotypes have different capsid proteins that determine their cell- and tissue-type tropism. These tropisms may differ across species. We have highlighted some of the limitations of wildtype serotypes and the lack of efficient systemic neurotropic capsids in section "AAVs enable gene delivery and circuit interrogation", paragraph 4, sentences 1-7. Our manuscript now reads:

> "However, these AAVs, including AAV9, have limitations that have prevented their wider use. For instance, their cell-type tropism can vary across species. In neonatal mice and macaques, intravenously administered AAV9 transduces neurons preferentially, whereas in juvenile and adult mice and macaques, the tropism shifts toward astrocytes (Bevan et al., 2011; Dehay et al., 2012; Foust et al., 2009; Gray et al., 2011; Mattar et al., 2013; Samaranch et al., 2012). Moreover, AAV9 and the other BBB crossing serotypes mentioned above have a higher tropism for peripheral organs such as the liver than the brain (Gray et al, 2011, Zincarelli et al, 2008). This is especially concerning in large animals such as NHPs as they require large volumes of virus for systemic delivery and the high doses of AAV needed to achieve clinical relevance can lead to hepatotoxicity or sensory neuron toxicity (Hinderer et al., 2018). Additionally, humans as well as NHPs harbor neutralizing anti-AAV antibodies to certain wildtype AAV serotypes from pre-existing exposure or develop anti-AAV antibodies after therapeutic rAAV administration (Louise Jeune et al, 2013). This is a limiting factor for gene therapy applications where subsequent viral administration may be needed if the transgene expression wanes over time."

R2Comment9: *This paragraph aims to provide a rationale why do we need NHP models of human diseases, and why we need better AAVs to achieve better models of human diseases… however it never really provides any arguments that the current models are not sufficient*

We have removed this paragraph for clarification. We have integrated relevant parts in other sections of the manuscript.

3rd paragraph

R2Comment10: *This paragraph gives an overview of different species of monkey used in research. It seems very disconnected from the narrative. It also makes statements like e.g. "Old world monkeys are useful models of cognition, attention, and memory, among other." But the authors never say why are they good models?*

We have rewritten this paragraph to reflect these concerns and better elaborate the importance of NHP models over rodent models. This paragraph now reads:

"Perhaps the most notable distinction between human and rodent brains is the expansion of neocortex during human evolution (Kaas, 2012; Öngür & Price, 2000; Petrides & Pandya, 1999, 2002; Pine et al., 2021). This expansion is often thought to have contributed to the many high order abilities and social complexities related to human uniqueness (Kaas, 2012; Smaers et al., 2011). Many studies in humans have shed light on the neuronal circuits associated with these abilities, however, due to the limitations of the available tools for the study of the human brain, a more comprehensive understanding of the underlying biology is still needed (Craig, 2009; Cristofori et al., 2019; Gläscher et al., 2012; Horga et al., 2014). To this

end, NHPs are of particular importance. To briefly review, NHPs can be roughly broken down into various simian species, which include monkeys and apes, and prosimians, such as lemurs. Monkeys can be further divided into Old World (Catarrhini) and New World (Platyrrhini) monkeys (Welker, 2017). Marmosets (*Callithrix jacchus*), which diverged from the human lineage approximately 35 million years ago (MYA), and rhesus macaques (*Macaca mulatta),* which diverged from humans even more recently, approximately ~25 MYA (FIG1), are the two most common NHPs used in research. It is this recent evolutionary divergence from a common ancestor that has made NHPs a valuable model in neuroscience, as they possess a highly elaborated prefrontal cortex, including a well-developed internal granular layer (Bernardi & Salzman, 2019; Öngür & Price, 2000; Petrides & Pandya, 1999, 2002). Because they are our phylogenetic neighbors, NHPs share many behavioral and anatomical features with humans (Kalin & Shelton, 2003; Öngür & Price, 2000; Petrides & Pandya, 1999, 2002; Phillips et al., 2014). For example, the ability to navigate social complexities has been hypothesized to be enabled by the evolutionary expansion of the primate prefrontal cortex((Dunbar & Shultz, 2007; Pine et al., 2021). Indeed, unlike many animals, both NHPs and humans have developed complex social behaviors that have helped them navigate the complexities of living in large social groups (Chang & Platt, 2014). These include prosocial behaviors (Miller et al., 2016), social imitation (Subiaul et al., 2004), and in New World Monkeys like marmosets, monogamy and infant rearing (Miller et al., 2016; Saito, 2015). This shared social repertoires between monkeys and humans has been helpful in studying the underlying biology of social behaviors (Chang & Platt, 2014; Ziegler, 2018). In addition to social behaviors, the phylogenetic proximity of Old World monkeys, like rhesus macaques, to humans provides an avenue to study the primate brain which has a similar structure and cytoarchitecture to the human brain (Öngür & Price, 2000; Petrides & Pandya, 1999, 2002). For this reason, primates can contribute to understanding human-specific cognitive functions like high order cognition, attention, and working memory (Brady & Hampton, 2018; Deaner & Platt, 2003; Dezfouli et al., 2021; Rich & Wallis, 2016; A. C. Snyder et al., 2021; Xie et al., 2022)."

4th paragraph

R2Comment11: *This paragraph describes the differences between the rodent and NHP brain physiology. Although this is a good paragraph to have in section dedicated to describing why NHP are valuable model of study human diseases, it is hard to see how it relates to the rest of the section. Furthermore, the authors focus on specific to NHP neuronal cells, however, the focus of this review are not AAV that target specific cells, but rather AAVs that are capable of infecting all brain*

While this review highlights novel engineered AAV capsids that are capable of infecting all brain, we mention under "Cell-type specific targeting using AAVs" that these capsids were tested using the ubiquitous CAG promoter which drives transgene expression in most cells and that replacing this promoter for a cell-type specific enhancer or promoter can restrict expression to select cell-types. In addition to Section "Cell-type specific targeting using AAVs", we have made further edits throughout the manuscript to clarify that neuronal dissection in NHPs requires both neurotropic capsids and the ability to target specific neuronal cell-types.

Section "AAV-based approaches for targeting specific cell-types in NHPs", Paragraph 1

"Developing tools to target specific cell-types to study their role in normal and disease circuitry remains a major challenge for primate neuroscience. Rodent models often rely on genetically-engineered Cre lines to achieve cell-type specificity. Unfortunately, primate gestational and maturational timelines preclude the widespread use of these genetic engineering approaches in primates (though see: (Drummer et al., 2021; Park et al., 2016; Sasaki et al., 2009; Tomioka et al., 2017)) so a different approach is needed. It is unlikely that capsid engineering alone will achieve the level of cell-type specificity required for NHP neuroscience research. However, profiling AAV capsid variants generated by selection experiments is time-consuming and labor-intensive, and most remain uncharacterized (Zolotukhin & Vandenberghe, 2022). Although new molecular and computational tools, such as machine learning, might facilitate capsid profiling, these approaches also have limitations (Zolotukhin & Vandenberghe, 2022). Additionally, studies in NHPs suggest that novel transduction properties may not only arise from unique capsid binding properties, but also from uncharacterized capsid-promoter interactions (Bohlen et al., 2020). Therefore, it is likely that achieving cell-type specificity in NHPs will depend on a combination of BBB-crossing AAV capsid variants and regulatory elements."

Section "Nonhuman primate as a valuable model for study of human diseases"

, Paragraph 4 , Sentence 5 :

"In this review, we highlight the need for more efficient neurotropic AAVs that can be delivered systemically in NHPs, recently engineered capsid variants that can cross the blood brain barrier in NHPs, and advances made to target specific cell-types."

Section "**AAVs enable gene delivery and circuit interrogation**"**, Paragraph 4:**

"When designing a study, it is important to take these considerations into account. In studies of the primate brain, it is important to ensure that the target gene sequence is reliably expressed, to minimize off-target effects, and to ensure animal safety. This has led most studies to prefer AAVs. However, if the genetic cargo is larger than optimal for an AAV genome, researchers run the risk of lower transduction efficiency, affecting their ability to perform the desired manipulation (Wu et al, 2010). These cost-benefit calculations are study-specific and constantly changing. Development of cell-type specificity, as a function of the viral capsid or shortened enhancer and promoter cargo, as described below could mitigate off-target effect. In the following sections, we will discuss current efforts to develop novel systemic rAAV vectors with high transduction efficiency and optimized biodistribution, with minimal off-target delivery, and low immunogenicity for gene delivery to the NHP CNS (Davidson et al, 2022, Challis et al, 2022, Y. A. Chan & Deverman, 2022)."

R2Comment12: *Overall this section needs major revisions. The paragraphs seem disconnected, and the message is somehow scattered. It needs better organization. After reading this paragraph, the reader still has hard time to give specific examples of why NHP are good models for human disease*

We have rewritten the section for clarity. Our edits are listed in R2's Comments 10 and 11. In addition, we have edited Paragraph 3, Sentences 1-2, to elaborate on the translational utility of NHPs for modeling human disease.

“While the phylogenetic proximity of NHPs to humans have made them anatomically and behaviorally similar, it is also likely that throughout evolution, the composition and function of neuronal circuits have adapted based on the evolutionary pressures placed on the species (Katz & Harris-Warrick, 1999). That is to say, while rodents and humans may share basic organization of circuits, changes within these circuits can cause large and important changes in behaviors (Katz & Harris-Warrick, 1999).”

Advantages of AAVs for circuit interrogation and gene delivery

R2Comment13: *First paragraph needs citations. The authors throw a lot of information with no citations…. This is not acceptable / scholarly*

We have updated to include the following references: Liu et al., 2022; Hui et al., 2022, Kristensson et al., 1982; Davidson and Breakefield, 2003

R2Comment14: *Although this section is titled "Advantages of AAVs for circuit interrogation and gene delivery" the authors only mention one AAV serotype, never talk about other viral vectors, and why would AAV be better than others…*

We have rewritten this section to mention other viral vectors as well as why AAVs are preferred. This change is reflected in Section “AAVs enable gene delivery and circuit interrogation” Paragraph 1, sentences 2-11:

“Viral vectors such as herpes simplex virus (HSV), rabies, adenovirus, lentivirus (LV), and adeno-associated viruses (AAVs) have emerged as an effective tool for neuroscience in that they enable neuronal tracing and functional interrogation through the delivery of various transgenes (Liu et al, 2022, Hui et al, 2022, Ghosh et al, 2020, Kristensson et al, 1982, Davidson and Breakefield, 2003). Viral vectors are composed of: i) a capsid, an outer protein shell enclosing the genetic material and which determines the vector’s tropism, or ability to infect different cell-types; ii) regulatory elements such as enhancers or promoters which restrict expression to specific cell or tissue types; and iii) a transgene (Bulcha et al, 2021). Transgenes include fluorescent proteins as genetic reporters for visualization, sensors for measuring neurotransmitter release (e.g., GCaMP, DLight, etc.), opsins and synthetic receptors for cellular manipulation (e.g., ChR2, DREADDs), and repair templates for CRISPR-Cas9 based gene editing and expression manipulation (e.g., using CRISPRa/i) (Yim et al, 2020, Patriarchi et al, 2018, Roth et al, 2016, Boyden et al, 2015, Klapoetke et al, 2014, Magnus et al, 2011, Zhang et al, 2007, Li et al, 2005). Such genetic tools have advanced our understanding of how various cell-types and specific circuits contribute to adaptive behaviors and emergent properties of the brain. Among the viral vectors, AAVs are considered to be the safest since they are non-pathogenic, and are naturally replication deficient i.e. they lack the genes necessary for replication and replicate only when co-infected with a helper virus (Rose and Koczot, 1972, Buller et al, 1981). In contrast, lentiviruses transduce cells with higher efficiency than AAVs but there is uncertainty surrounding their safety due to the possibility of random insertional mutagenesis (Zheng et al., 2018). This can affect the genetic code at the DNA insertion site, leading to adverse outcomes, including cancer (Zheng et al., 2018). Similarly, herpes viral vectors can cause strong inflammatory responses (Ghosh et al, 2020). These non-specific and adverse effects have precluded them from widespread use in NHPs (Trembley et al.,2020). Additionally, AAVs are capable of inducing stable, long-term transgene expression in both dividing

> and post-mitotic cells such as neurons, making them ideally suited for gene manipulation studies that require stable expression in cells that have already matured (Bartlett et al, 2008). For these reasons, recombinant AAVs (rAAVs) have become the viral vector of choice for *in vivo* gene therapy applications, with more than 285 registered clinical trials to date (Kuzmin et al, 2021, U.S. National Library of Medicine (www.clinicaltrials.gov)."

R2Comment15: *Inconsistency among different parts of the paper. E.g. this section starts with "The ability to define, monitor, and manipulate a neural circuit requires precise delivery of reporters… (…)" the AAVs mentioned in the graphical abstract do not address the problem of specificity, but rather provide brain-wide expression*

We have edited our graphical abstract to highlight future directions which includes combining our engineered AAVs with enhancers and promotors to target specific neuronal populations in the brain. Furthermore, our reasoning behind the graphical abstract is addressed in a previous comment (R2Comment2). Additionally, our goal in this review was to introduce novel AAV variants that can bypass the BBB better than commonly used AAV9 or AAV.PHP.eB. We are, however, aware that neural circuit dissection requires precise delivery of genes of interest. While genetic CRE lines have made cell type specificity a possibility in rodent models, unfortunately, this is not the norm in NHP research. Because of this, it is likely that cell type specificity in NHPs will be achieved through a combination of both capsid and DNA regulatory elements. This is discussed further in the following section "AAV-based approaches for targeting specific cell-types in NHPs" (Reviewer2Comment11).

R2Comment16: *"Additionally, AAVs are capable of transducing both dividing and non-dividing cells with stable, long-term transgene expression in post-mitotic cells"*

Why is that important?

We have edited the manuscript to clarify the importance of long term transgene expression. This change is reflected in Section "AAVs enable gene delivery and circuit interrogation" Paragraph 1, sentences 11-13:

> "Additionally, AAVs are capable of inducing stable, long-term transgene expression in both dividing and post-mitotic cells such as neurons, making them ideally suited for gene manipulation studies that require stable expression in cells that have already matured (Bartlett et al, 2008)."

R2Comment17: *Citation?*

We have updated to include a reference to Bartlett et al, 2008.

R2Comment18: *How does is compare to other viral vectors?*

We have included in our manuscript some limitations of AAVs. Please see response to comment above (R2Comment14). This change is reflected in Section "AAVs enable gene delivery and circuit interrogation".

R2Comment19: *Why is that important for NEURO?*

We have added additional information to clarify why this is important for neuroscience. This change is reflected in Section "AAVs enable gene delivery and circuit interrogation", paragraph 5. Our manuscript now reads:

“When designing a study, it is important to take these considerations into account. In studies of the primate brain, it is important to ensure that the target gene sequence is reliably expressed, to minimize off-target effects, and to ensure animal safety. This has led most studies to prefer AAVs. However, if the genetic cargo is larger than optimal for an AAV genome, researchers run the risk of lower transduction efficiency, affecting their ability to perform the desired manipulation (Wu et al., 2010). These cost-benefit calculations are study-specific and constantly changing. Development of cell-type specificity, as a function of the viral capsid or shortened enhancer and promoter cargo, as described below could mitigate off-target effect. In the following sections, we will discuss current efforts to develop novel systemic rAAV vectors with high transduction efficiency and optimized biodistribution, with minimal off-target delivery, and low immunogenicity for gene delivery to the NHP CNS (R. C. Challis et al., 2022; Y. A. Chan & Deverman, 2022; Davidson et al., 2022).”

"For the study and treatment of neurological and neurodegenerative diseases such as Parkinson-s disease, widespread transgene expression within the CNS is desired"

R2Comment20: *Citation?*

We have included references to Sun and Roy, 2021, Hadaczek et al, 2016, Muramatsu et al, 2010

R2Comment21: *Why is it important to have a widespread transgene expression?*

We have rewritten for better clarity. This change is reflected in Section “AAVs enable gene delivery and circuit interrogation”, paragraph 2, sentences 1-15:

“For the study and treatment of neurological and neurodegenerative diseases, widespread distribution of transgene expression could be transformative (Sun and Roy, 2021, Hadaczek et al, 2016, Muramatsu et al, 2010). For example, idiopathic Parkinson’s disease is hypothesized to result from the aggregation of a protein called alpha-synuclein (α-Syn) first in the enteric nervous system, before it propagates up the vagus nerve to the basal forebrain, midbrain and ultimately the cerebral cortex (Braak et al, 2003). rAAVs can be used to deliver a pathogenic protein such as α-Syn to model PD in animals, and help tease apart the cell-types in the ENS and CNS that are susceptible to α-Syn pathology (Alam et al, 2022, Huntington and Srinivasan, 2021, Ulusoy et al, 2010, Kirik and Bjorklund, 2003, Challis et al, 2020). In such cases, widespread transgene expression is required. Conversely, the pathogenic protein can be silenced, or the disease phenotype may be reversed by delivering a therapeutic gene such as *GBA1*, which encodes the lysosomal enzyme Glucerebrosidase, and has been shown to reduce inflammation and aggregation of α-Syn in models of PD as well as Gaucher’s disease- a lysosomal neurodegenerative disorder (Bjorklund et al, 2021, Sardi et al, 2013, Sardi et al, 2011). The route of AAV administration, dose, age at the time of injection, and preexisting neutralizing antibodies against it in the host, are all key determinants of an AAV’s safety, efficacy and tropism (Y. A. Chan & Deverman, 2022). There are many advantages to direct in-brain injection. In fact, most studies to date have directly injected viruses into the brain to deliver genetic cargo to specific regions. This has been performed in animal models of PD to target the putamen or substantia nigra (Bartus et al, 2013 , Kells et al, 2012, Muramatsu et al, 2010, Christine et al, 2009, Kaplitt et al, 2007). Despite its many advantages, which include relatively dense and robust expression surrounding the

> infusion site, targeting large, diffuse, or spatially distributed regions can require multiple injections. Thus, this route of administration is most suitable for localized targets. Covering entire brain regions remains a challenge due to the size of the primate brain and often requires multiple craniotomies (Wang, 2021,). Systemic delivery of AAVs obviates the need for multiple direct injections and importantly reduces the health risks associated with extremely long and highly invasive surgeries. As a therapeutic approach, systemic administration via a single injection might be a safer alternative toward achieving brain wide gene transduction (Kimura and Harashima, 2022, Bourdenx et al, 2014). Ultimately, increased efficacy of BBB-crossing AAVs may be combined with other technologies to achieve localized expression, as we suggest below."

R2Comment22: *Studying how?*

Please see the response above to R2Comment21.

R2Comment23: *How using the AAV to study PD? To model the disease?*

Please see the response above to R2Comment21.

R2Comment24:*I would argue that for treatment we need specificity not widespread expression within the CNS. We have widespread effects of the systemic drugs e.g. LDopa, and the WIDESPREAD nature of its action is in fact problematic. Focused, specific treatment is much more desired*

We agree that widespread transgene gene expression across the CNS is not necessary for all therapeutic approaches and have modified paragraph 2 under this section. However, even achieving sufficient coverage of brain regions such as striatum for AAV-mediated PD treatment through local injections is difficult due to the size of the primate brain and can cause tissue damage due to the surgery's invasiveness. Please see R2Comment1.

R2Comment25: *"Thus, systemically-delivered AAV variants that can transduce neurons with higher efficacy are still needed for NHP studies"*

The authors only brought up the AAV9… there are multiple other serotypes that transduce neurons

While other AAV serotypes do indeed transduce cells, only AAV9 is able to transduce the CNS via systemic delivery. Other natural AAV serotypes have not been shown to bypass the BBB efficiently, thus we focused on AAV9. We have further elaborated on this in Section "AAVs enable gene delivery and circuit interrogation" Paragraph 3-4:

> "To date, 12 distinct naturally-occurring or *wildtype* serotypes of AAVs, (AAV1-12) have been identified in humans and NHPs (Agbandje-McKenna & Kleinschmidt, 2012).. Each of these serotypes differs in capsid structure and, therefore, tropism (Agbandje-McKenna & Kleinschmidt, 2012). The most commonly used serotypes in rodent research are AAV2, AAV5, AAV8, and AAV9, which transduce the CNS, although some transduce other organs as well (Aschauer et al., 2013). In NHPs, the most commonly used serotypes are AAV5 and AAV9 (Tremblay et al., 2020). AAV9 has been particularly widely studied because of its ability to cross the BBB and has been employed in several CNS-targeted gene therapies (Y. A. Chan & Deverman, 2022; W. Chen et al., 2021; Foust et al., 2009; Song et al., 2022; B. Yang et al., 2014; H. Zhang et al., 2011). Efforts have also been made to characterize other serotypes that also have the capability to cross the BBB. For example,

Gao et al. cloned and identified more than 100 novel rAAVs from human and NHP tissues (G. Gao et al., 2005; G. Gao et al., 2002). Among these, AAVrh8, AAVrh10 and AAVhu32 were found to cross the BBB with high efficiencies, similar to AAV9.

However, these AAVs, including AAV9, have limitations that have prevented their wider use. For instance, their cell-type tropism can vary across species. In neonatal mice and macaques, intravenously administered AAV9 transduces neurons preferentially, whereas in juvenile and adult mice and macaques, the tropism shifts toward astrocytes (Bevan et al., 2011; Dehay et al., 2012; Foust et al., 2009; Gray et al., 2011; Mattar et al., 2013; Samaranch et al., 2012). Moreover, AAV9 and the other BBB crossing serotypes mentioned above have a higher tropism for peripheral organs such as the liver than the brain (Gray et al, 2011, Zincarelli et al, 2008). This is especially concerning in large animals such as NHPs as they require large volumes of virus for systemic delivery and the high doses of AAV needed to achieve clinical relevance can lead to hepatotoxicity or sensory neuron toxicity (Hinderer et al., 2018). Additionally, humans as well as NHPs harbor neutralizing anti-AAV antibodies to certain wildtype AAV serotypes from pre-existing exposure or develop anti-AAV antibodies after therapeutic rAAV administration (Louise Jeune et al, 2013). This is a limiting factor for gene therapy applications where subsequent viral administration may be needed if the transgene expression wanes over time."

R2Comment26: *This section is titled "Advantages of AAVs for circuit interrogation and gene delivery", however I do not think that the content follows. There is only one sentence that might provide some exemplary characteristic of AAVs that give them advantages over OTHER viral vectors, however the authors have never even mentioned other viral vectors*

What are other viral vectors?

Please see the response above to R2Comment14.

R2Comment27: *Why AAVs are better suited for NHP studies than e.g. lenti virus?*

Please see the response above to R2Comment14.

R2Comment28: *What characteristics of viral vectors are desired for NHP studies?*

Please see response above to R2Comment19.

R2Comment29: *What are serotypes?*

We have included more detail about AAV serotypes in our manuscript. Please see the responses above to R2Comment6, R2Comment7 and R2Comment25.

R2Comment30: *What AAV serotypes are there?*

Please see the responses above to R2Comment6, R2Comment7 and R2Comment25.

R2Comment31: *Why AAVs are better for circuit interrogation than other viral vectors?*

Please see response above to R2Comment14.

Capsid engineering for systemic delivery to achieve tissue specific biodistribution in NHPs

R2Comment32: *This section is practically the only one that is related to the graphical abstract.*

This is intentional. A number of the other sections are largely reviewing the need for these tools. We believe the field is better suited by a graphical abstract that targets these specific advances, rather than the rationale for why they are important. We have clarified in the revised manuscript.

Although it does describe the artificially engineered capsids, it fails to provide crucial information to the reader e.g.:

R2Comment33: *How does capsid and capsid modification relate to tropism?*

We have edited the manuscript to clarify how capsid and capsid modification relates to tropism. This change is reflected in Section "Capsid engineering for systemic delivery to achieve tissue specific biodistribution in NHPs", Paragraph 1:

> "The capsid of an AAV is its primary point of interaction with receptors on the host cell surface which enable the virus to be internalized, and ultimately deliver their genetic cargo to the cell nucleus (R. C. Challis et al., 2022; C. Li & Samulski, 2020). Because of this, the capsid structure of AAVs have been widely researched in order to determine the protein domains responsible for cellular receptor binding, and consequently the virus' tropism and efficacy (R. C. Challis et al., 2022; E. J. Lee et al., 2018; C. Li & Samulski, 2020). Capsid modification or engineering is one route toward altering an AAV's tropism and efficacy as several permissive sites for rational and random amino acid substitutions and insertion have been identified (R. C. Challis et al., 2022). Through capsid engineering, we can enhance and refine AAV tropisms, as well as identify novel AAV serotypes with improved BBB crossing properties."

R2Comment34: *What is the capsid? What does it do?*

We have clarified what a capsid is and its role in the biology of an AAV. This change is in Section "AAVs enable gene delivery and circuit interrogation", Paragraph 1, sentence 3:

> "Viral vectors are composed of: i) a capsid, an outer protein shell enclosing the genetic material and which determines the vector's tropism, or ability to infect different cell-types; ii) regulatory elements such as enhancers or promoters which restrict expression to specific cell or tissue types; and iii) a transgene (Bulcha et al, 2021)."

R2Comment35: *Why does the capsid matter for the viral vectors?*

We have edited the manuscript to state why capsids matter for the viral vector. This change is reflected in section "Capsid engineering for systemic delivery to achieve tissue specific biodistribution in NHPs", Paragraph 1, sentence 1:

"The capsid of an AAV is its primary point of interaction with receptors on the host cell surface which enable the virus to be internalized, and ultimately deliver their genetic cargo to the cell nucleus (R. C. Challis et al., 2022; C. Li & Samulski, 2020)."

R2Comment36: *How do capsids and serotypes relate?*

We have elaborated on the relationship between capsids and serotypes. This change is reflected in section "AAVs enable gene delivery and circuit interrogation", paragraph 3, sentences 1-2:

> "To date, 12 distinct naturally-occurring or *wildtype* serotypes of AAVs, (AAV1-12) have been identified in humans and NHPs (Agbandje-McKenna and Kleinschmidt, 2011). Each of these serotypes differs in capsid structure and, therefore, tropism (Agbandje-McKenna and Kleinschmidt, 2011)."

R2Comment37: *How do capsid affect tropism and efficacy?*

Please see the response above to R2Comment33.

R2Comment38: *What types of capsids are there already?*

We have mentioned some of our engineered capsids- AAV-PhP.B, AAV-PhP.eB, AAV-CAP-B10, AAV-CAP-B22, AAV-MaCPNS1, AAV-MaCPNS2, AAV-CAP-Mac. A complete list of capsids is outside the scope of this review but can be found in the references provided in Section "Capsid engineering for systemic delivery to achieve tissue specific biodistribution in NHPs".

Cell-type specific targeting

R2Comment39: *rAAV have not been only tested in NHP, but have been used in variety of studies involving optogenetics, neuroanatomical labeling…*

it is not clear how capsid design relate to cell-specificity… in the example of the rAAVs it is not per se the capsid design as the injection site that specifies the cell type that is transduced… yes the retro nature of this viral capsid allows for the retrograded uptake, but the cell specificity is not achieved in this manner… e.g. if you inject rAAV to SC you will express the genes carried by this viral vector in all (except GABAergic cells which for unknown reason do not work with rAAVs) projections to the SC… so there is not cell specificity

We apologize for the confusion. For clarity, we have moved this sentence.

R2Comment40: *Why are promoters and enhancers important?*

We have elaborated on the importance of promoters and enhancers in Paragraph 2. This change is reflected in Section "AAV-based approaches for targeting specific cell-types in NHPs", Paragraph 2:

> "Cis- acting regulatory DNA elements, such as promoters and enhancers, are sequences of DNA that proteins bind to in order to initiate and increase the likelihood of transcription respectively (Wittkopp et al, 2011, Levine, 2010). Thus promoters and enhancers can determine the level of transgene expression and the cells they are expressed in. Ubiquitous promoters, such as cytomegalovirus (CMV), chicken β-actin (CBA), human elongation factor 1 alpha (EF1α) or combinations of these such as CMV early enhancer/chicken beta actin (CAG), drive high levels of transgene expression in most cell-types (Haery et al, 2019). However, high, widespread transgene expression is not always desired and can evoke immune responses to the transgene product (Perez et al, 2020, Samelson-Jones et al, 2020). Alternatively, cell-type specific promoters can be incorporated into the AAV cargo. These can be used, for instance, to target neurons (synapsin 1) or astrocytes (glial fibrillary acidic protein), or even more specifically dopaminergic neurons,

cerebellar Purkinje cells, or parvalbumin (PVALB) neurons in the brain (El-Shamayleh et al., 2017; Hoshino et al., 2021; Matsuzaki et al., 2014; Nitta et al., 2017; Shinohara et al., 2016; Stauffer et al., 2016, Boulos et al, 2006). Promoter sizes can range anywhere from ~100 bp to 1000 bp (Domenger and Grimm, 2019). Due to the AAV's size limitation, ongoing efforts are focused on identifying shorter, and phylogenetically conserved, regulatory element sequences to direct cell-type specific transgene expression across species (Domenger and Grimm, 2019, de Leeuw et al, 2016, Matsuzaki et al, 2014, Nathanson et al, 2009)."

R2Comment41: *What do enhancers and promoters do?*

Please see the response above to R2Comment40.

R2Comment42: *How do enhancers and promoters could achieve cell-specificity?*

Please see the response above to R2Comment40.

R2Comment43: *"Single-cell and single nucleus transcriptomics studies of the rodent and primate brain have revealed the molecular complexity and diversity of cell types present". How do single-cell and single-nucleus transcriptomics and the molecular complexity that they reveal, relate to the cell-specificity of the AAV?*

We have reworded this section for clarity. This change is now reflected in Section "AAV-based approaches for targeting specific cell-types in NHPs", Paragraph 4, Sentences 1-3:

> **"**Single-cell and single-nucleus transcriptomics studies of the rodent and primate brain have revealed the molecular complexity and diversity of cell-types present based on their gene expression profiles (Hodge et al., 2019; Tasic et al., 2016, 2018; Zeisel et al., 2015). In the primary motor cortex alone, there are potentially 45 conserved cell-types among mouse, marmoset and human (BICCN, 2021). Only once a cell-type has been molecularly defined can researchers begin to identify DNA regulatory elements that are required for cell-type specific gene activation, and guide the development of tailored targeting strategies for intervention and functional interrogation."

R2Comment44: *How do these studies can help creating new enhancers and promoters?*

In brief, single cell- or -nucleus sequencing studies help us with the identification of molecularly distinct cell-types present in a particular region (eg. cortex) and marker genes i.e. genes that are highly enriched in those cell-types. Then with experiments such as ATAC-seq (Assay for Transposase-Accessible Chromatin with high-throughput sequencing), we can profile the open chromatin regions next to these marker genes. DNA regulatory elements bind to these open chromatin regions and dictate gene transcription. Moreover, chromatin accessibility varies by cell-type as well as tissue-type. Thus it is important to know the pattern of DNA regulatory elements important for the transcription/activation of cell-type specific marker genes in order to efficiently target these cell-types and understand their role in normal and disease circuitry. Please see response to R2Comment43. Additionally, we have included citations in Section "AAV-based approaches for targeting specific cell-types in NHPs", paragraph 2, sentence 1, of studies that discuss this process in more detail:

> **“**Recently, chromatin profiling techniques coupled with next-generation sequencing have led to the discovery of putative enhancers that are less than 600 bp, and can drive cell-type specific activation of genes (Grandi et al, 2022, Buenrostro et al, 2015, Cusanovich et al., 2015; Fang et al., 2021; Graybuck et al., 2021; Hrvatin et al., 2019; Mich et al., 2021; Nair et al., 2020; Preissl et al., 2018; Rubin et al., 2020; Visel et al., 2013; Vormstein-Schneider et al., 2020).”

"Importantly, these enhancers tend to be smaller in size in comparison to promoters, allowing for more flexibility with AAV payload which is limited to 4.7kb"

R2Comment45: *Lack of citation *

This sentence has been reworded and we have updated it to include references to Domenger and Grimm, 2019, de Leeuw et al, 2016, Matsuzaki et al, 2014, Nathanson et al, 2009. This change is now reflected in Section “AAV-based approaches for targeting specific cell-types in NHPs”, Paragraph 2, Sentence 10:

> **“**Due to the AAV’s size limitation, there are ongoing efforts to optimize the AAV cargo to target specific cell-types conserved across species, by identifying shorter regulatory element sequences that are phylogenetically conserved (Domenger and Grimm, 2019, de Leeuw et al, 2016, Matsuzaki et al, 2014, Nathanson et al, 2009).”

R2Comment46: *Why is the payload important?*

Please see the response above to R2Comment19.

R2Comment47: *What is the difference between enhancers and promoters?*

Please see the response above to R2Comment40.

R2Comment48: *Why is small size of the enhancers important?*

This is addressed in Section “AAV-based approaches for targeting specific cell-types in NHPs”, Paragraph 2, Sentence 8:

> “Due to the AAV’s size limitation, ongoing efforts are focused on identifying shorter, and phylogenetically conserved, regulatory element sequences to direct cell-type specific transgene expression across species (Domenger and Grimm, 2019, de Leeuw et al, 2016, Matsuzaki et al, 2014, Nathanson et al, 2009).”

"AAV-PHP.eB containing hDLX2.0 upstream of minimal beta-globin promoter and super yellow fluorescent protein-2 (SYFP2) reporter reportedly transduced GABAergic interneurons ex vivo (…)"

R2Comment49: *Lack of citation*

We have included the reference to Mich et al, 2021.

"Combinatorial methods involving dual vector injections with distinct enhancers and promoter elements have also been attempted to target certain cell-types"

R2Comment50: *what are these "combinatorial methods"?*

This sentence has been reworded for better understanding. This change is now reflected in Section "AAV-based approaches for targeting specific cell-types in NHPs", Paragraph 4, Sentences 5-27

> "These variants, which show comparatively higher neuronal transduction than AAV9 in NHPs, can be used to screen regulatory elements that specifically target neuronal subpopulations. One caveat of this approach is that injecting multiple AAVs with different enhancer and promoter elements in the same animal may cause interference between the regulatory elements, resulting in a loss of specificity compared to independent delivery (Pouchelon et al, 2022, Mehta et al, 2019). This may confound interpretation of pooled screens of putative regulatory elements."

R2Comment51: *Not clear what is this method and how it relates to cell specificity…*

Please see the response above to R2Comment50.

R2Comment52: *The title of this section implies that the authors will discuss variety of cell types within the brain and how they can be targeted using specific AAV serotypes and/or enhancers and promoters. However, the authors never mentioned all the classes and subclasses of the neurons that are there… what cells are conserved between species and what are not? and what are the ones that are specific for NHP? (they only give few examples)*

Citations have been provided which discuss all the classes and sub-classes of neurons in the CNS, as well as cell classes that are conserved or not across species. An in-depth list of cell-types is outside the scope of the review. Additionally the title of this section has been changed to "AAV-based approaches for targeting specific cell-types in NHPs".

R2Comment53: *Do the enhancers that work for the different classes in rodents work the same in NHP?*

Even when the enhancer sequence is conserved, enhancers tested in mice cannot be assumed to maintain cell-type specificity or function the same way in primates. This must be tested. We have clarified and provided examples of how orthologous sequences of the *Dlx5/6* enhancer and PV enhancers have been tested *in vivo* in mouse and macaque with comparable levels of specificity. This change is now reflected in Section "AAV-based approaches for targeting specific cell-types in NHPs", Paragraph 3, Sentences 2-9:

> " A distal-less homeobox (*Dlx*) gene enhancer sequence that targets GABAergic interneurons in the telencephalon of several vertebrate species including mouse and marmoset was identified (Dimidschstein et al., 2016; A. T. Lee et al., 2014, Zerucha et al, 2000). Additionally, the mouse ortholog of the *Dlx5/6* enhancer (mDLX5/6), which is only ~400 bp, packaged into either AAV1 or AAV9, showed similar specificity for GABAergic interneurons when locally injected into area V1 of the primary visual cortex of rhesus macaques (De et al., 2020). Mich et al further optimized the human ortholog of the *Dlx5/6* enhancer (hDLXI5/6i) by engineering a triple tandem of core elements taken from hDLXI5/6i and called it hDLX2.0 (Mich et al., 2021). AAV-PHP.eB containing hDLX2.0 upstream of a minimal beta-globin promoter and super yellow fluorescent protein-2 (SYFP2) reporter transduced GABAergic interneurons in *ex vivo Macaca nemestrina* cortical slice cultures and human neocortical slice cultures (Mich et al, 2021). Putative enhancers for targeting PVALB-expressing interneurons have similarly been identified, packaged in AAV-PHP.eB and tested in mice via retro-orbital injections, and in marmoset and macaque via local or intraparenchymal injections (Lawler et al., 2022; Mich et al., 2021; Vormstein-Schneider et al.,

2020). These enhancers either targeted PVALB interneurons broadly or specific sub-classes of PVALB interneurons in the neocortex in both mouse and NHP. To identify regulatory elements that can drive faithful expression across species using AAV vectors, the selection method has largely focused on sequences that are conserved across species. However, Mich et al reported that certain PVALB enhancer sequences present in the open chromatin analyses of the human neocortex but not in the mouse neocortex, were still able to drive selective expression in PVALB neurons in the mouse brain (Mich et al, 2021, Vormstein-Schneider et al., 2020). Thus, to minimize the number of experimental animals used for *in vivo* screening, it may be advantageous to develop machine-learning classifiers that can identify DNA sequence patterns important for driving species-agnostic cell-type specific activation (Lawler et al, 2022)."

Discussion

R2Comment54: *The discussion focuses on summarizing the few scatter information about the three artificially engineered AAVs. As a result it seems disconnected from the whole narrative.*

We have renamed the Discussion to "Future Directions" and edited the manuscript to be more clear and connected to the narrative. This section now reads:

"The effective delivery of sensors, effectors, and reporters for circuit tracing and manipulation, largely depends on the vector used. However, differences in brain size and immune function have hampered the widespread adoption of genetic technology in monkeys. Thus, the engineering of more efficient and specific viral vectors to target the CNS in NHPs addresses many of the challenges that have inhibited progress in translating rodent disease biology to better therapies and therapeutic approaches. Here, we review new engineered systemic capsid variants that address these challenges. Specifically, using an adapted, cross-species directed evolution approach, our group has identified new variants that can transduce neuronal cells in CNS and PNS via peripheral injection in multiple NHP species commonly used in research.

In marmosets, AAV-CAP-B10 and AAV-CAP-B22, variants of AAV-PHP.eB, were identified to have enhanced CNS transduction compared to AAV9. In both marmosets and rhesus macaques, AAV-MaCPNS1 and AAV-MaCPNS2 variants transduced cells in both CNS and PNS. This may be particularly useful for studies where widespread transgene expression is desired as infusions in NHPs typically require multiple sites of injection to cover whole areas of tissue; however, this remains to be tested in relation to distributed brain function and/or behavior. In both rhesus macaques and green monkeys, AAV-CAP-Mac was found to transduce a higher percentage of neurons than AAV9. Importantly, these variants show significantly lower transduction in the liver compared to AAV9, thereby minimizing the risk of hepatotoxicity.

Still, while these novel AAVs may provide a new and necessary tool for delivering genetic cargo into the primate brain, many technical issues still remain. Below we briefly discuss these issues.

1. *Achieving cell-type specificity in NHPs*. For decades, studies in NHPs have relied on lesions, reversible inactivation, and electrophysiology to elucidate the role of specific regions in a particular function (Balan et al., 2019; Dal Monte et al., 2015; Lak et al., 2014; Rudebeck et al., 2013). While this has provided invaluable insight into distributed circuits

that underlie behavior, these studies are limited by their cell-type agnostic nature. Lesioning and reversible inactivation generally impact all cells in a particular region. In addition, lesion studies have resulted in conflicting reports on observed behaviors within the same region of the brain, often due to unintended damage to fibers of passage (Rudebeck et al., 2013). Similarly, electrophysiological recording techniques cannot differentiate molecular cell-types, and rely on electrophysiological-specific characterization (e.g. early-firing, late-firing, ramping, etc.). For example, in the ventral tegmental area, a minority of neurons share the same electrophysiological properties as dopamine neurons, leading to questions on whether some recordings have been misattributed to dopamine (Ungless & Grace, 2012). To address this issue, rodent studies have used TH-Cre mice to target dopamine-expressing VTA neurons for the expression of sensors and effectors (Bariselli et al., 2016; Lindeberg et al., 2004). However, in NHPs, Cre-lines do not yet exist or are not widely used. It is unlikely that capsid engineering alone will enable cell-type specificity . Instead,it is likely that a combination of capsid and DNA regulatory elements will achieve cell-type specificity. To this end, AAV-Cap-Mac and AAV-MaCPNS1/2 can be used to screen regulatory elements that specifically target neuronal subpopulations.

2. *Effective delivery and functional efficacy of genetic cargo.* Advances in genetic cargo, like opsins and DREADDs, have been critical in dissecting circuits that are thought to contribute to disease -- in rodents. However, these techniques have not been widely adopted in NHPs because of difficulties in delivering genetic cargo efficiently into the primate brain. Currently, only a few published studies in NHPs have demonstrated successful delivery of opsins, with AAV5 and AAV9 being amongst the most commonly used vectors (Tremblay et al., 2020). Moreover, outcome measures- anatomy, physiology, and behavior in these studies-vary greatly (Bliss-Moreau et al., 2022). For example, AAV5 has been shown to preferentially target some brain regions but not others (Roseboom et al., 2021). Still, a major hurdle remains in determining the extent in which systemic delivery can express effector-cargo in a sufficient proportion of cells to affect behavior. Even so, infecting a small proportion of cells can still help us better understand the contributions of a small number of cells on behaviors as the functional efficacy of sensor-cargo does not necessitate a large proportion of cells. Currently, the effective delivery and functional relevance of sensors and effectors have not been tested using AAV-CAP-Mac or AAV-MaCPNS1/2. Further work is needed to show the functional efficacy of the genetic cargo delivered by these novel AAVs.

Understanding the emergence or origins of brain-based disease requires coordinated cross-species research. To this end, NHP models are particularly important because of their shared biology with humans. However, tools for interrogating anatomical pathways and functional circuits will need to be translated for widespread use in NHPs. While the vectors presented here address many common technical challenges seen in NHPs, there is a continued need for more efficient and specific AAVs. We hope that further optimization of these vectors can lead to more efficient delivery and ultimately, lead to new tools for the study of the primate brain and the development of new treatments for brain-based disorders.”

Accept Letter

Dear Dr. Campos,

Thank you for submitting your manuscript to the Special Issue in Current Research in Neurobiology entitled **Illuminating the Monkey Brain: Organization, Networks and Circuits.** I am pleased to inform you that your manuscript has been accepted for publication. A few minor reviewer comments, are appended below.

Your accepted manuscript will now be transferred to our production department. We will create a proof which you will be asked to check, and you will also be asked to complete a number of online forms required for publication. If we need additional information from you during the production process, we will contact you directly.

We appreciate and value your contribution to Current Research in Neurobiology. We regularly invite authors of recently published manuscript to participate in the peer review process. If you were not already part of the journal's reviewer pool, you have now been added to it. We look forward to your continued participation in our journal, and we hope you will consider us again for future submissions.

CRNEUR aims to be a unique, community-led journal, as highlighted in the Editorial Introduction. As part of this vision, we will be regularly seeking input from the scientific community and encourage you and your co-authors to take the survey.

We would also like to invite you to take part in our CRNEUR Author Question & Answer (Q&A), which could get published alongside your article and help to promote it. We suspect you might have an interesting story of perseverance or team work that was required for the research study to complete, or a diversity of perspectives that you might share, as a way of inspiring others about neuroscience.

Kind regards,

Yogita Chudasama
Associate Editor
Current Research in Neurobiology

Reviewer comments:

Reviewer 1: Two minor grammatical catches, otherwise I am happy with the changes the authors have made and recommend publication.

1. This shared social repertoires between monkeys and humans has been helpful in studying the underlying biology of social behaviors - I think the authors should change "This" to "The", perhaps?

2. To date, 12 distinct naturally-occurring or wildtype serotypes of AAVs, (AAV1-12) have been identified in humans and NHPs (Agbandje-McKenna & Kleinschmidt, 2012).. - Remove the second period

Reviewer 2: I was glad to see that the authors have addressed my comments and concerns very carefully

and thoroughly. The use of the AAV technology in NHP opens up new and exciting possibilities reaching out from answering the basic question on how the brain works to development of new, target specific treatments. I think this review will serve as an important source of information for the scientific community.

-------- *End of Review Comments* --------
